# Comparison of the effect of 360° versus two-dimensional virtual reality video on history taking and physical examination skills learning among undergraduate medical students: a randomized controlled trial

**DOI:** 10.1007/s10055-022-00664-0

**Published:** 2022-08-16

**Authors:** Yi-Ping Chao, Chung-Jan Kang, Hai-Hua Chuang, Ming-Ju Hsieh, Yu-Che Chang, Terry B. J. Kuo, Cheryl C. H. Yang, Chung-Guei Huang, Tuan-Jen Fang, Hsueh-Yu Li, Li-Ang Lee

**Affiliations:** 1 Department of Computer Science and Information Engineering, Graduate Institute of Medical Mechatronics, Chang Gung University, 33302 Taoyuan, Taiwan; 2 Department of Neurology, Chang Gung Memorial Hospital, Linkou Main Branch, 33305 Taoyuan, Taiwan; 3 Faculty of Medicine, Graduate Institute of Clinical Medicine Sciences, College of Medicine, Chang Gung University, 33302 Taoyuan, Taiwan; 4 Department of Otorhinolaryngology-Head and Neck Surgery, Chang Gung Memorial Hospital, No. 5, Fu-Hsing Street, Gueishan District, Linkou Main Branch, 33305 Taoyuan, Taiwan, Republic of China; 5 Department of Family Medicine, Chang Gung Memorial Hospital, Linkou Main Branch, Taoyuan, 33305 Taiwan; 6 School of Medicine, College of Life Science, National Tsing Hua University, Hsinchu, 300044 Taiwan; 7 Department of Industrial Engineering and Management, National Taipei University of Technology, 10608 Taipei, Taiwan; 8 Department of Surgery, Chang Gung Memorial Hospital, Linkou Main Branch, Taoyuan, 33305 Taiwan; 9 Department of Emergency Medicine, Chang Gung Memorial Hospital, Linkou Main Branch, Taoyuan, 33305 Taiwan; 10 Institute of Brain Science, National Yang Ming Chiao Tung University, 11221 Taipei, Taiwan; 11 Department of Laboratory Medicine, Chang Gung Memorial Hospital, Linkou Main Branch, 33305 Taoyuan, Taiwan; 12 Department of Medical Biotechnology and Laboratory Science, Graduate Institute of Biomedical Sciences, Chang Gung University, 33302 Taoyuan, Taiwan

**Keywords:** Cognitive load, History taking, Physical examination, Two-dimensional video, Virtual reality, 360° video

## Abstract

Before caring for patients, video instruction is commonly used for undergraduate medical students, and 360° virtual reality (VR) videos have gained increasing interest in clinical medical education. Therefore, the effect of immersive 360° VR video learning compared with two-dimensional (2D) VR video learning in clinical skills acquisition should be evaluated. This randomized, intervention-controlled clinical trial was aimed to assess whether immersive 360° VR video improves undergraduate medical students' learning effectiveness and reduces the cognitive load in history taking and physical examination (H&P) training. From May 1 2018 to October 30 2018, 64 senior undergraduate medical students in a tertiary academic hospital were randomized to receive a 10-min immersive 360° (360° VR video group; *n* = 32) or 2D VR instructional video (2D VR video group; *n* = 32), including essential knowledge and competency of H&P. The demographic characteristics of the two groups were comparable for age, sex, and cognitive style. The total procedure skill score, physical examination score, learner’s satisfaction score, and total cognitive load in the 360° VR video group were significantly higher than those in the 2D VR video group (effect sizes [95% confidence interval]: 0.72 [0.21–1.22], 0.63 [0.12–1.13], 0.56 [0.06–1.06], and 0.53 [0.03–1.03], respectively). This study suggested that a10-minute 360° VR video instruction helped undergraduate medical students perform fundamental H&P skills as effectively as 2D VR video. Furthermore, the 360° VR video might result in significantly better procedural metrics of physical examinations with higher learner satisfaction despite the higher cognitive load.

**Supplementary Information:**

The online version contains supplementary material available at 10.1007/s10055-022-00664-0.

## Introduction

History taking and physical examination (H&P) are critical core competencies of undergraduate medical education (UME). Performing H&P can deepen the relationship between students and patients, direct clinical reasoning, and lead to further assessments for a differential diagnosis (von Fragstein et al. 2008). Therefore, H&P instruction is essential for UME students to learn how to provide comprehensive care to patients (Noble, Scott-Smith, O'Neill, Salisbury, & Education 2018). In addition to traditional instructional methods such as scripts, classroom lectures, and faculty teaching in a clinical setting to learn basic H&P knowledge and skills, UME students can also use modern learning methods such as standardized patients, videos, e-learning, small group workshops, simulations, and virtual reality (VR) to improve these core competencies (Danielson et al. 2019; Keifenheim et al. 2015; Lee et al. 2018a, b, 2018a; Letterie 2002).

VR can create vivid memories and emotionally engaging experiences (Riva et al. 2007). There has been a large volume of research on VR applications in an educational context (Abich et al. 2021), e.g., medical knowledge, examinations, communication, procedures, treatment, and surgery (Checa, Miguel-Alonso, & Bustillo, 2021; Chen et al. 2021; Lohre et al. 2020; Nas et al. 2020; Wu et al. 2021). Immersive VR learning can target a high level of simulation fidelity (Lungu et al. 2021) and impose a low level of cognitive load (Andersen et al. 2016b), which is beneficial when applying this education to the real world. Recently, 360° VR videos have been used to create practical, highly immersive, three-dimensional (3D) educational systems (Izard, et al. 2018; Pulijala et al. 2018; Yoganathan et al. 2018). In these systems, learners can watch a 360° VR video from a first-person perspective at a time of their choosing. Notably, a first-person perspective has been shown to improve learning outcomes (Fiorella et al. 2017).

Recent evidence-based studies have demonstrated that VR instruction is effective for teaching and assessments (Alaker et al. 2016; Kyaw et al. 2019). For example, medical students using immersive VR learning have a higher knowledge gain than non-immersive VR learning (Gutiérrez et al 2007). Furthermore, immersive VR training allows the learners to perform clinical techniques (such as administering the anesthesia) more accurately and confidently (Collaço et al. 2021). Moreover, VR learning has been shown to help acquire complex skills outside the classroom due to reduced working hours, fewer learning sessions, and patient safety issues (Alaker, et al. 2016). Although the effect of the first-person perspective in 360° VR video may be more robust for complex tasks regarding accuracy and time, it may not be as effective as imitating actions while learning or explaining how to perform H&P skills during clinical assessments (Fiorella et al. 2017). Some medical educators have highlighted the importance of improving the current evidence level of VR-based learning (Khan et al. 2018). Thus, further studies with a high level of evidence for the effect of 360° VR video on learning outcomes are needed.

VR instructional applications need to apply cognitive load theory to reduce cognitive load to improve learning and skill acquisition (Andersen et al. 2016b). For example, VR learning with enhancing cognitive load has been shown to improve the performance of a surgical task compared to solitary VR learning (Sankaranarayanan et al. 2020). In contrast, immersive VR learning, which induces a higher cognitive load, has been associated with a more unsatisfactory performance than conventional VR learning (Frederiksen et al. 2020). However, the cognitive load depends on the user’s prior experience and self-efficacy within the learning environment (Vasile et al. 2011). Therefore, evaluating the cognitive load in VR-based learning is of increasing interest for medical education.

The global COVID-19 pandemic is having an enormous impact on medical education. With medical institutions temporarily closed, lectures switched to online, clinical rotations delayed, clinical skill evaluations compromised or even canceled (Tariq et al. 2020), in-person educational activities have been significantly reduced for infection control. Consequently, virtual pedagogical activities for teaching H&P skills (Sukumar et al. 2021) are increasing. VR is one of the most effective direct instructions (Lee, Lim, Jeon, Song 2020). However, the effects of 360º video, compared with two-dimensional (2D) video, through a VR headset (and more generally immersive versus non-immersive VR) on measures of learning and cognitive load are not well understood.

### The present study

This study aimed to compare the effects of 360° VR video learning with 2D VR video learning on H&P skills, cognitive load, and learning satisfaction in the real world using a randomized controlled trial with allocation concealment, blinding of outcome assessment, and intention-to-treat analysis. For achieving these purposes, both VR videos' source recording was the same and came from a 360º camera held by the physician. Then the investigators offered the student the possibility to have a full 360º experience via a VR headset and controllers, with a first-person perspective but de-coupled from that of the physician, or to have a limited experience in which the field of view was restricted to 120º and the video perspective was entirely controlled by the physician and not by the student in a 2D theater-like projection.

#### Hypothesis

The null hypothesis of this study was that "360° VR video improves learner's H&P performance and cognitive load in the same way as 2D VR video".

## Methods

### Ethical considerations

We conducted this prospective, intervention-controlled clinical trial (Supplement 1) from August 1 2017 to July 31 2020 at an academic teaching hospital (Department of Otorhinolaryngology, Head and Neck Surgery [ORL-HNS], Linkou Chang Gung Memorial Hospital, Taoyuan, Taiwan). This study was approved by the Institutional Review Board of Chang Gung Medical Foundation (No: 201601821B0), and all procedures were conducted in compliance with the Declaration of Helsinki 1975. All the participants were informed about the study aims and provided written informed consent. The study followed the Consolidated Standards of Reporting Trials (CONSORT) guidelines (Moher et al. 2010).

### Participants

**Fig. 1:**
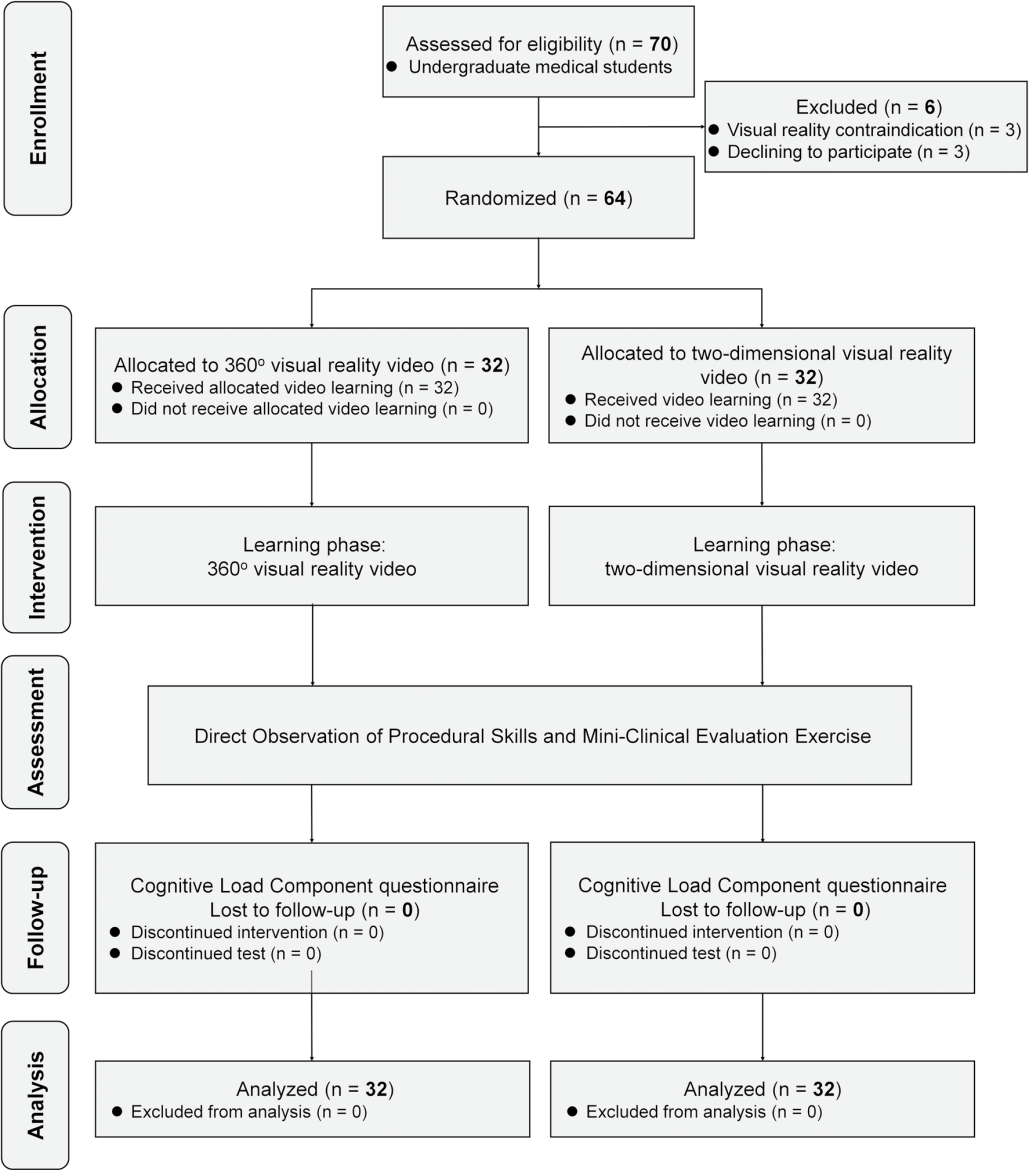
CONSORT flow diagram

We invited final-year UME students older than 20 years and novices of ORL-HNS from our department to participate. The exclusion criteria were: (1) contraindications for VR such as recent motion sickness, heart conditions, epileptic symptoms, and (2) refusing to participate. One of the authors (Lee LA) held information meetings about the study. All participants had been shown the practical aspects of using VR headsets and controllers and confirmed to have the essential ability to use the head-mounted display and controllers. Furthermore, the cognitive style of each learner was determined using the Group Embedded Figures Test (GEFT) (Lee et al. 2018a, b; Scott et al. 1981): “field-dependence” (GEFT score ≤ 12) and “field-independence” (GEFT score > 12) (Witkin et al. 1971). The study flowchart is shown in Fig. 1.

### Randomization and blinding

According to the literature, age (Plancher et al. 2010), sex (Tippett et al. 2009), and cognitive style (Lee et al. 2018a, b; Scott et al. 1981) were covariates of new educational technology such as VR and mobile e-learning. For controlling sample size and covariates, the stratified randomization method was applied (Suresh KP. 2011). The Random Number Generator in IBM SPSS software (version 25; IBM, Armonk, NY, USA) was used to create a list of random numbers to allocate the students with a fixed block size of 8 in parallel groups. After the participants had been identified and assigned into blocks (matched for age, sex, and cognitive style), we performed the simple randomization within each block to allocate the participants (1:1) to a 360° VR video group and 2D video group. The allocation sequence was concealed before the implementation of the video module.

### Instructional materials of video learning

**Fig. 2:**
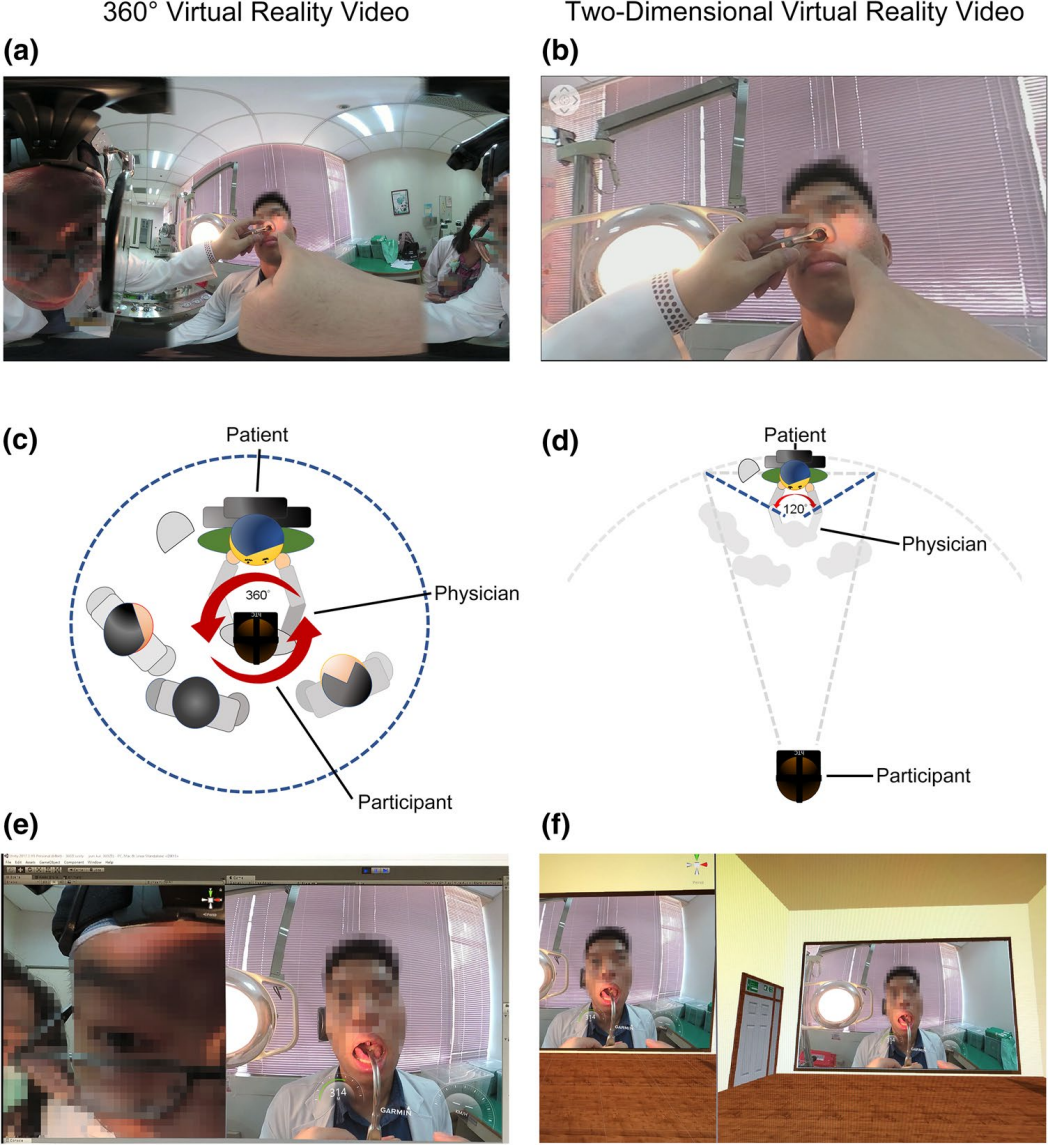
Differences in original video **a**, **b**, field of view **c**, **d**, and perspective of the software platform **e**, **f** between 360° and two-dimensional virtual reality videos

A 10-min instructional video (video encoder: H264/Advanced Video Coding; resolution: 3840 pixels × 2160 pixels; framerate: 30 frames/s), including essential knowledge and procedural skills of H&P according to the guidelines of the American Board of Otolaryngology (Tsue 2014), was made with analysis, design, development, implementation, and evaluation models (Morrison et al. 2013) (Supplement 2). Briefly, the video showed what the learners needed to know about H&P skills using multimedia demonstrations of different worked examples (Renkl et al. 1998) with self-explanation prompts (Chi et al. 1989). The source recording for both the 360º and the alternative experience was the same and came from a 360º camera (Garmin VIRB 360, Garmin Ltd., Kansas City, MO, United States) held by the physician. The 360° VR video provided a 360° view (Fig. 2a) and the 2D VR video (Fig. 2b) provided a 120° view from the camera’s perspective. One physician (filmmaker, located in the middle of the gray character group; Fig. 2c and d) introduced procedures of classical H&P and performed a physical examination on a virtual patient (the colored character). The immersive 360**°** VR video (Fig. 2a) was created to play through a VR head-mounted display so that the participants could explore the video at 360° arbitrarily (via a VR headset and controllers), with a first-person perspective but de-coupled from that of the physician (Fig. 2c). In contrast, the participants could watch the 2D VR video controlled by the filmmaker in a 120° focused field of view (Fig. 2b) through the same head-mounted display (Fig. 2d). Therefore, the 2D VR video participants observed the scene "from the eyes of the physician." From the software platform, the 360° VR video provided a direct first-person perspective (Fig. 2e), while the 2D VR video was displayed in a theater environment to provide a third-person perspective (Fig. 2f). Two experienced instructors (Chuang HH & Kang CJ) verified that the 360° and 2D VR videos contained similar learning materials. The main differences between both modules included visual angle (360° versus 120°), immersion (immersive versus non-immersive), and perspective (first person versus third person) (Chao et al. 2021). Courseware with the same user interface of the 360°and 2D VR video modules was then developed using Unity 2017.3.1 Editor (Unity Technologies, San Francisco, CA, USA) by two investigators (Chao YP & Lee LA).

### Intervention

After randomization, all participants were given 10 min to watch their allocated VR video (either 360° or 2D) through a VR headset (Vive VR headset, HTC Corp., New Taipei, Taiwan).

In the 360° VR video group, the learners could watch and listen to the instructor’s demonstrations of H&P skills and the responses of a simulated patient and other medical staff from a first-person perspective at a time of their choosing (Fig. 2e).

In the 2D VR video group, the learners simply watched and listened to the instructor’s demonstrations and the responses of a simulated patient and other medical staff from a third-person perspective (Fig. 2f).

### Methods of measurement

Within 60 min after the intervention, each learner conducted a focused H&P with a real outpatient in a teaching clinic for 10 to 20 min. We used the Direct Observation of Procedural Skills (DOPS) to measure physical examination skills, the Mini-CEX to measure generic H&P skills, and the Cognitive Load Component (CLC) questionnaire to measure cognitive load.

#### Direct observation of procedural skills

Each participant performed a real-patient H&P for a maximum of 20 min. Two separate investigators (Fang TJ & Lee LA), blinded to video group allocation, used a DOPS (J. Norcini & Burch 2007; Yang et al. 2011) rating form to assess the procedural skills of the participant for ORL-HNS physical examinations. In this study, we used a ten-item teacher-scored DOPS form to rate ten behaviors, using a ten-point rating scale (below expectations = 1–2; borderline for completion = 3–5; meets expectations = 6–8; and above expectations = 9–10; not observed = blank) (Table 1). The DOPS has been shown to have acceptable reliability (Cronbach's alpha = 0.70–0.80; intraclass correlation coefficient = 0.25–0.85) and validity (predictive validity coefficient = 0.38–0.51) across different standardized measures (Erfani Khanghahi & Ebadi Fard Azar 2018; Kara et al. 2018).

**Table 1:** Comparison of procedural skills using the Direct Observation of Procedural Skills (DOPS)

Outcomes	360° virtual reality video group (*n* = 32)	Two-dimensional virtual reality video group (*n* = 32)	Mean Difference (95% confidence interval)	*P*-value
DOPS-total: total score	88.4 (4.0)	85.8 (3.2)	0.7 (.2−1.2)	.01
DOPS-1: demonstrating an understanding of indications, relevant anatomy and procedural techniques	9.0 (0.6)	8.7 (0.7)	0.5 (−0.04−1.0)	.14
DOPS-2: explaining and obtaining agreement	9.0 (0.8)	8.8 (0.7)	0.3 (−0.2−0.8)	.21
DOPS-3: demonstrating appropriate preparation pre-procedure	9.1 (0.8)	8.7 (0.6)	0.6 (0.1−1.1)	.03
DOPS-4: proper determination of the examination areas	8.9 (0.6)	8.4 (0.8)	0.7 (0.2−1.2)	.01
DOPS-5: technical ability to perform skill safely	8.8 (0.7)	8.2 (0.8)	0.8 (0.3−1.3)	.002
DOPS-6: aseptic technique	8.7 (0.7)	8.6 (0.6)	0.2 (−0.3−0.6)	.45
DOPS-7: seeking help when appropriate	8.8 (0.7)	8.5 (0.6)	0.5 (−0.04−1.0)	.06
DOPS-8: post procedure management	8.7 (0.6)	8.6 (0.5)	0.2 (−0.3−0.7)	.65
DOPS-9: communication skills, consideration of the patient, and professionalism	8.7 (0.5)	8.6 (0.5)	0.2 (−0.3−0.7)	.34
DOPS-10: overall performance	8.8 (0.4)	8.7 (0.5)	0.2 (−0.3−0.7)	.15
Data are expressed as mean (standard deviation). The DOPS-total (range = 10–100) is defined by the sum of ten items, using a ten-point rating scale				

#### Mini-clinical evaluation exercise

When each participant performed a real-patient H&P, the same investigators (Fang TJ & Lee LA) assessed the participants’ competencies using a Mini-CEX rating form (Table 2) (Chang et al. 2013; J. J. Norcini 1995). The Mini-CEX has been shown to have good reliability (Cronbach's alpha = 0.75; intraclass correlation coefficient = 0.78) (Eggleton et al. 2016) and a predictive validity coefficient ranging from 0.26 to 0.86 across different standardized measures (Al Ansari, Ali, & Donnon 2013). The rating form contained seven clinical competencies using a nine-point rating scale (1 = unsatisfactory and 9 = superior; blank = not observed). The results were also assessed for the satisfaction of both the assessor and learner using a nine-point rating scale (1 = unsatisfactory and 9 = superior) (Chang, et al. 2013; Kogan et al. 2002).

**Table 2:** Comparison of procedural skills using the Mini-Clinical Evaluation Exercise (Mini-CEX)

Outcomes	360° virtual reality video group (*n* = 32)	Two-dimensional virtual reality video group (*n* = 32)	Mean Difference (95% confidence interval)	*P*-value
Mini-CEX-total: total score	40.1 (4.1)	39.8 (5.2)	0.1 (−0.4−0.6)	.75
Mini-CEX-1: medical interview	5.8 (0.6)	5.9 (0.7)	−0.2 (−0.6–0.3)	.58
Mini-CEX-2: physical examination	5.3 (0.8)	4.8 (0.8)	0.6 (0.1–1.1)	.02
Mini-CEX-3: professionalism	5.9 (0.7)	5.8 (0.8)	0.1 (−0.4–0.6)	.52
Mini-CEX-4: clinical judgment	5.5 (0.6)	5.6 (1.0)	−0.1 (−0.6–0.4)	.76
Mini-CEX-5: counseling skills	5.9 (0.8)	5.8 (0.9)	0.1 (−0.4–0.6)	.55
Mini-CEX-6: organization/efficiency	5.7 (0.7)	5.8 (0.9)	−0.1 (−0.6–0.4)	.54
Mini-CEX-7: overall clinical competence	6.0 (0.5)	6.0 (0.8)	0 (−0.5–0.5)	.89
Assessor satisfaction	8.9 (0.3)	8.8 (0.4)	0.3 (−0.2–0.8)	.46
Learner satisfaction	8.9 (0.3)	8.6 (0.7)	0.6 (0.1–1.1)	.02
Data are expressed as mean (standard deviation). The Mini-CEX-total (range = 7–63) is defined by the sum of seven items, using a nine-point rating scale, and the assessor and learner satisfaction, using a nine-point rating scale				

#### The cognitive load component questionnaire

The CLC questionnaire includes six items that are used to measure intrinsic (task difficulty and complexity), extraneous (instructional clarity and relevance), and germane (practical focus and amount of learning) cognitive loads (Naismith et al. 2015a, b). The participants rated the level of each item using a five-point Likert scale (1 = not at all and 5 = extremely) (Table 3). Therefore, the score of each type of cognitive load was calculated as the sum of the two specific scales (range = 2–10), and the total cognitive load score was calculated as the sum of all six scales (range = 6–30). The CLC questionnaire has been shown to have moderate correlations with different standardized measures such as the Paas Cognitive Load Scale (Paas F. G. 1992) and National Aeronautics and Space Administration Task Load Index (Hart and Staveland 1988) (Pearson correlation coefficient = 0.40–0.62) (Naismith et al. 2015a, b) and acceptable reliability (Cronbach's alpha = 0.71) (Toy et al. 2020). The validity of CLC questionnaire scores as a psychometric measure has also been shown in workshop designs and evaluations (Naismith et al. 2015a, b).

**Table 3:** Comparison of cognitive load using the Cognitive Load Component (CLC) questionnaire

Outcomes	360° virtual reality video group (*n* = 32)	Two-dimensional virtual reality video group (*n* = 32)	Mean Difference (95% confidence interval)	*P*-value
CLC-total	20.1 (2.0)	18.9 (2.5)	0.5 (0.03–1.0)	.04
Intrinsic cognitive load	4.7 (1.6)	3.7 (1.4)	0.7 (0.2–1.2)	.01
CLC-1: How difficult did you find the simulation session?	2.4 (1.0)	1.8 (0.8)	0.7 (0.2–1.2)	.04
CLC-2: How complex was the content covered in the simulation session?	2.3 (0.8)	1.9 (0.7)	0.4 (−0.001–0.8)	.05
Extraneous cognitive load	7.5 (1.1)	7.5 (1.1)	−0.3 (−0.5–0.03)	.82
CLC-3: How clear did you find the instructions for the simulation session?	3.8 (0.6)	4.0 (0.5)	0.1 (−0.5–0.03)	.08
CLC-4: How relevant did you find the simulation session for your current practice?	3.7 (0.8)	3.5 (1.0)	0.2 (−0.3–0.6)	.40
Germane cognitive load	7.8 (0.8)	7.7 (1.4)	0.09 (−0.40–0.58)	.67
CLC-5: How focused were you during the simulation session?	3.9 (0.7)	3.7 (0.9)	0.2 (−0.2–0.6)	.36
CLC-6: How much did you learn from the simulation session?	3.9 (0.5)	4.0 (0.8)	−0.1 (−0.4–0.3)	.71
Data are expressed as mean (standard deviation). The CLC-total (range = 6–30) is defined by the sum of six items, using a five-point rating scale and the score of each type of cognitive load is calculated as the sum of the two specific scales (range = 2–10)				

### Outcome measures

The primary outcome measures of this study were the DOPS-total score and subscale scores after the intervention. The secondary outcomes included the Mini-CEX-total and subscale scores and CLC-total and subscale scores.

### Data analysis

Continuous data were reported as means (standard deviations [SD]) because all variables were normally distributed. Categorical variables were presented as numbers (percentages). A sample size of 32 participants per group was estimated using primary outcome effects (DOPS-total score) based on a priori study (360° VR video group = 90.2 [5.6] and 2D VR video group = 84.9 [5.1]) and an effect size of 0.99, type I error of 0.05, a power of 0.95, and 10–15% dropout rate. Sample size calculations were performed using G*Power 3.1.9.2 software (Heinrich-Heine University, Dusseldorf, Germany). Differences between groups were analyzed using the unpaired Student’s t-test or Fisher’s exact test as appropriate. All *P*-values were two‐sided, and statistical significance was accepted at *P* < 0.05. Statistical analyses were performed using GraphPad Prism for Windows version 9.0 (GraphPad Software Inc., San Diego, CA, USA).

## Results

### Participant characteristics

Seventy volunteers expressed interest, among whom six were excluded (Fig. 1). All of the 64 volunteers (mean age = 24.2 [0.9] years; 44 [69%] males and 20 [31%] females) who were enrolled (100%) completed the study. Baseline characteristics were comparable between the two groups. The mean (SD) age was 24.1 (0.8) years in the 360° VR video group and 24.4 (1.1) years in the 2D VR video group. There were 22 (69%) male students in the 360° VR video group and 22 (69%) in the 2D VR video group. Thirty (94%) students in the 360° VR video group and 30 (94%) in the 2D VR video group were classified as being field-independent.

### Primary outcomes

**Fig. 3:**
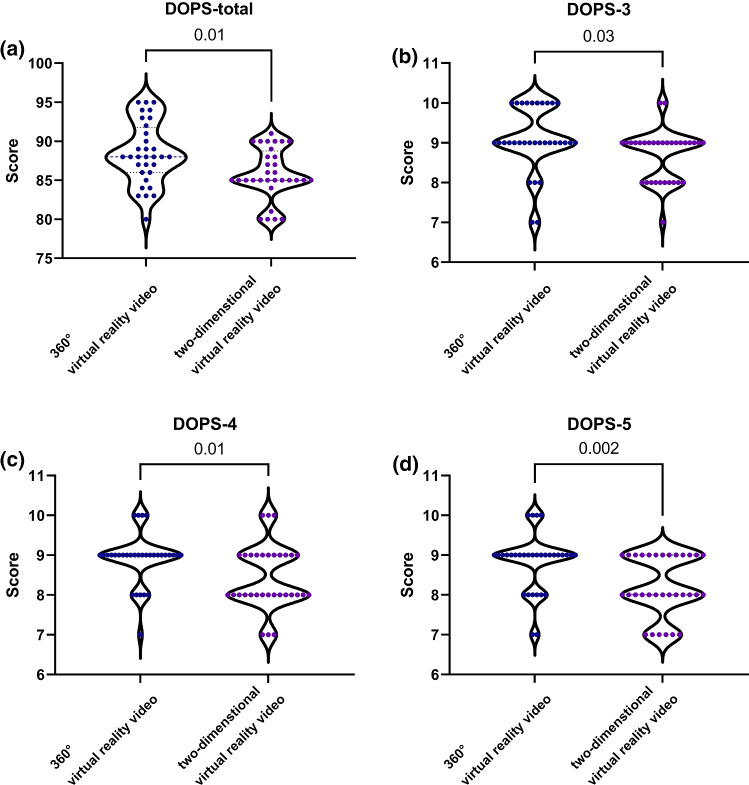
Violin plots demonstrating differences in the total and subscale scores of the Direct Observation of Procedural Skills (DOPS) between 360° and two-dimensional virtual reality videos: DOPS-total score **(a)**, DOPS-3 score **(b)**, DOPS-4 score **(c)**, and DOPS-5 score (**d**)

The mean DOPS-total score (88.4 [4.0]) in the 360° VR video group was significantly higher than that (85.8 [3.2]) in the 2D VR video group (effect size = 0.72) (Table 1; Fig. 3a). For the optimal assessment of physical examination skills, we further used three representative items (DOPS-3 [demonstrating appropriate preparation pre-procedure], DOPS-4 [proper determination of the examination areas], and DOPS-5 [technical ability to perform the skills safely]) which best reflected the observed student-patient interaction within this category. The mean skill DOPS-3 (Fig. 3b), DOPS-4 (Fig. 3c), and DOPS-5 (Fig. 3d) scores in the 360° VR video group were significantly higher than those in the 2D VR video group (effect sizes = 0.57, 0.71, and 0.80, respectively).

### Secondary outcomes

**Fig. 4:**
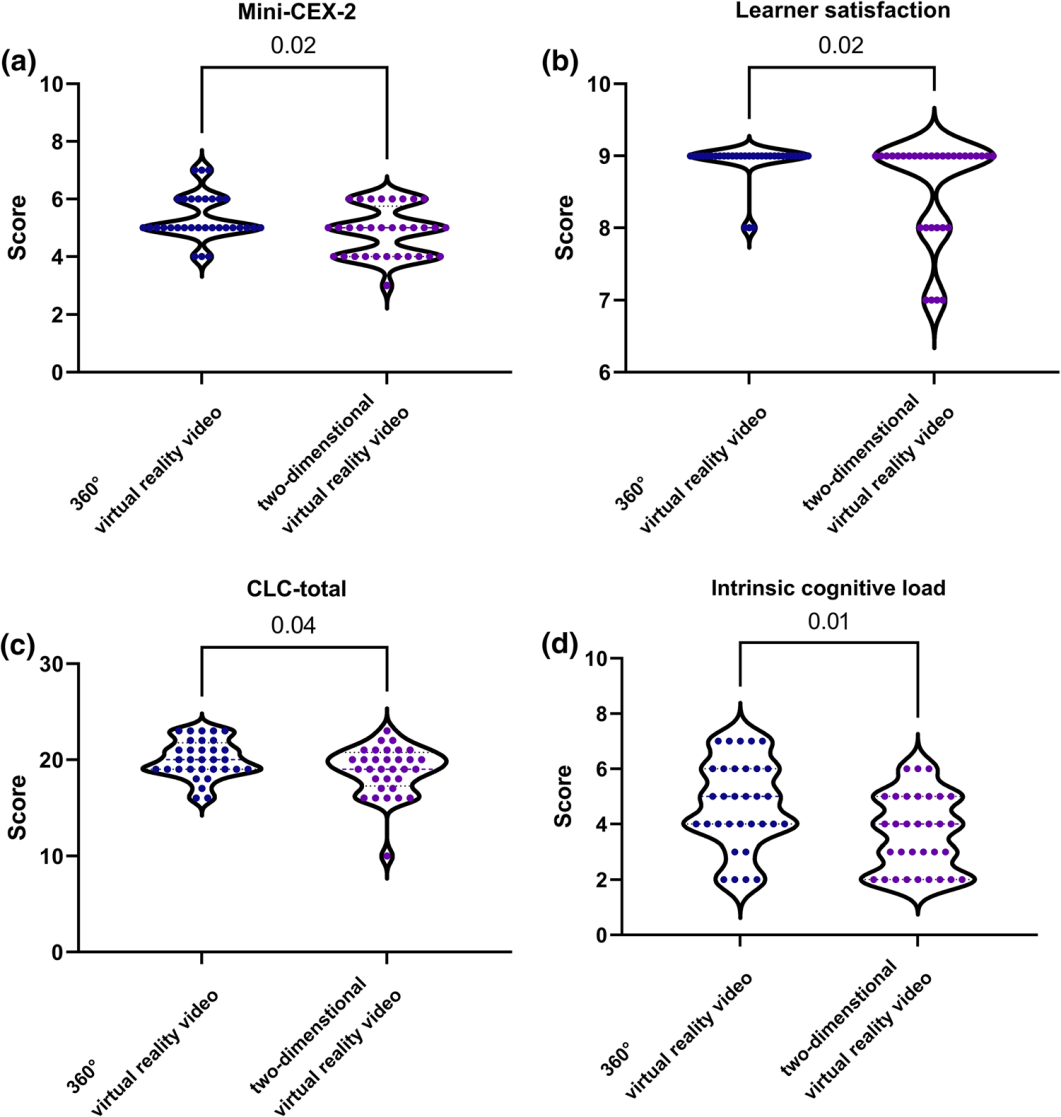
Violin plots demonstrating differences in the Mini-Clinical Evaluation Exercise (Mini-CEX)-2 score **(a)**, learner satisfaction **(b)**, the Cognitive Load Component (CLC)-total score **(c)**, and intrinsic cognitive load **(d)** between 360° and two-dimensional virtual reality videos

There was no significant difference in the mean Mini CEX-total score between the two groups (Table 2). Among the three most commonly encountered clinical competencies of H&P in ORL-HNS teaching clinics (Mini-CEX-1 [medical interview], Mini-CEX-2 [physical examination], Mini-CEX-5 [counseling skills]), only the mean Mini-CEX-2 score (5.3 [0.8]) in the 360° VR video group was significantly higher than that (4.8 [0.8]) in the 2D VR video group (effect size = 0.63) (Fig. 4a). Furthermore, the 360° VR video group had a significantly higher learner satisfaction score (8.9 [0.3]) than the 2D VR video group (8.6 [0.7]) (effect size = 0.56) (Fig. 4b).

The total (Fig. 4c) and intrinsic (Fig. 4d) cognitive load scores (20.1 [2.0] and 4.7 [1.6], respectively) in the 360° VR video group were significantly higher than those (18.9 [2.5] and 3.7 [1.4], respectively) in the 2D VR video group (effect sizes = 0.53 and 0.67, respectively). In contrast, differences in extraneous and germane cognitive load scores were not statistically significant.

## Discussion

To our knowledge, this is the first clinical trial to report the outcomes of using VR technology to educate novice UME students on performing fundamental H&P skills. Although the 360° VR video triggered higher total and intrinsic cognitive loads than the 2D VR video, it helped the learners perform physical examinations more readily, appropriately, and safely with higher self-satisfaction. In addition, the 360° VR video review was as effective as the 2D VR video review in learning how to perform a generic H&P skill as evaluated by the Mini-CEX assessment, even though these two models differed in content and perspective.

These findings support the use of 360° VR video to teach novice UME students essential H&P skills. Therefore, immersive 360° VR video with a higher field of view and degree of freedom is a cost-effective pedagogical tool by increasing learner attention, presence, skill enhancement, confident usability, performance, satisfaction, motivation, and engagement (Blair et al. 2021; Buttussi & Chittaro 2018). Notably, although the 360° VR video (i.e., immersive VR) was more effective in teaching clinical skills such as patient communication (Sultan et al. 2019), knot tying (Yoganathan et al. 2018), dental anesthesia (Collaço et al. 2021), procedure safety (Buttussi & Chittaro 2021), and fundamental H&P skills (Chao et al. 2021) than the 2D VR video (i.e., non-immersive VR), not all VR participants preferred immersive VR-based learning activity (Chao et al. 2021). In contrast, users of non-immersive VR (such as desktop-VR) preferred desktop-VR more for mine rescuing (Pedram et al. 2021) or had better knowledge gain for virtual biology learning (Makransky et al. 2019) than immersive 360° VR. Thereby, the suitability of VR technology to the learning task is more important than its advancement (Pedram et al. 2021).

Furthermore, the 360º VR learners may decide to look at something which is not relevant (whatever is happening behind the physician), while the 2D VR video learners can quickly focus on the relevant part of the scenario that the physician controls. In our preliminary study, the 360º VR learners watched the instructional scenes of physical examination with higher interest and engagement and longer secondary-task reaction time than the 2D VR video learners (Chao et al 2021). On the other hand, the 2D VR video learners emphasized that they found the 2D VR video module was easy to follow and highly efficient because the video was directed through the physician’s view. Therefore, each of the two VR video modules had its own advantages and disadvantages in H&P learning.

Moreover, creating 360° VR videos from a close to real-life perspective is crucial to engage the learner's interest without incurring high costs and computer programming obstacles. To reduce stimulator sickness's intensity, we developed a high-quality 360° VR application by using high-resolution (4 K) 360° videos, ergonomic VR software, and a high-end head-mounted display. After improving the quality of the 360° VR video, none of the pilot study subjects reported intolerable simulator sickness after experiencing a 10-min 360º VR video (Chao, et al. 2021). Furthermore, a pre-briefing session of the teaching clinic was usually less than 15 min; thereby, we considered a 10-min 360° VR video review was suitable for the H&P skill learning. In this study, the actual costs (work hours of computer programming) associated with the development of 360º VR video and 2D VR video instructional materials were approximate 25,000 USD (81 h) and 10,000 USD (54 h), respectively. However, the 360° VR video in this study did not increase the skill levels of history taking, counseling, and communication. We supposed that the self-explanation prompts (Chi et al. 1989; Hansen & Richland 2020) could elicit sophisticated self-explanations from the learners to boost deep learning and solve problems regardless of the video module. Nevertheless, relatively high medical interview and counseling skills (mean scores > 5.5) indicated that our participants had sufficient medical knowledge and communication skills regardless of which type of video learning.

Sultan et al*.* demonstrated that a pre-briefing session and a debriefing session between 360° VR video sessions could allow learners to reflect, give feedback to fill gaps, and enhance medical knowledge and communication skills (Sultan et al. 2019). Therefore, blended learning, including 360° VR video and face-to-face learning, may be able to enhance the competency of history taking to the next level of proficiency. Nevertheless, future studies are warranted to confirm the benefits of blended learning to educate UME students on performing H&P skills.

According to the cognitive load theory framework (Sweller 1988), an increase in intrinsic and extraneous cognitive loads in parallel with a decrease in germane cognitive load may explain the lower performance in video learning as reported with other multimedia. In laparoscopic training, immersive VR simulation training has been shown to trigger a higher cognitive load and contribute to worse performance than conventional VR simulation training. Therefore, we used worked examples (Lange et al. 2021; Renkl et al. 1998) to help the learners process the examples more deeply when learning with the videos. However, even though both video modules contained similar instructional content and used the same device to learn the H&P skills, the amount of mental effort involved in the 360° VR video was significantly higher than that involved in the 2D VR video (Frederiksen et al. 2020). Our preliminary study indicated that worked examples helped keep similar intrinsic cognitive load (assessed by the Paas Cognitive Load Scale and National Aeronautics and Space Administration Task Load Index) and mental efforts (evaluated by the National Aeronautics and Space Administration Task Load Index), but 360° VR video still triggered a higher physical demand than 2D VR video (Chao et al. 2021). However, we found the intrinsic cognitive load (assessed by the CLC questionnaire) of the 360° VR video group was significantly higher than that of the 2D VR video group in this study. Furthermore, we also found a higher physical demand subscale related to a higher level of simulator sickness while using the 360° VR application in the *post-hoc* study (Hsin LJ et al. 2022).

The increase in intrinsic cognitive load may be due to the complex virtual environment and additional medical staff responses. Hence, the high cognitive load may have hampered the learners’ performance (Sewell et al. 2019). However, the intrinsic cognitive load was relatively lower than the extraneous and germane cognitive loads in this study. According to the feedback we got from the participants with CLC questionnaires, the contents of both the 360° VR video and 2D VR video modules were a little bit more difficult and complex than expected, yet quite clear, relevant, and focused. Participants from both groups reported that they’d learned a lot from the program. Furthermore, the similar learning outcomes in both video groups suggest that the learners effectively increased cognitive function to process excessive information during the 360° VR video learning.

However, this randomized controlled trial indicated that a 10-min 360° VR video learning did not help the UME students perform history taking better and more satisfactorily than a 10-min 2D VR video learning. Notably, novices have been shown to use distributed VR practice better than massed VR practice to improve the learning curve (Andersen et al. 2015) and cognitive load (Andersen, Mikkelsen, Konge, Caye-Thomasen, & Sorensen, 2016a). Therefore, medical teachers should consider including 360° VR video in a competency-based learning curriculum to acquire H&P skills, eventually leading to improved quality of care.

Finally, age, sex, and cognitive style have been covariates of VR (Plancher et al. 2010; Tippett et al. 2009) and mobile e-learning (Lee et al. 2018a, b). For example, we found that both cognitive styles and learning modules were significantly associated with knowledge gain and satisfaction of mobile e-learning for emergent ORL-HNS disorders (Lee et al. 2018a, b). Therefore, we controlled the proportion of cognitive styles in the randomized allocation of participants. In the *post-hoc* analysis, age was significantly associated with Mini-CEX-total score and Mini-CEX-5 score (Pearson correlation test; r = 0.34 and 0.27, respectively; *P* = 0.01 and 0.03, respectively), whereas sex and cognitive style were not related to the DOPS-, Mini-CEX-, and CLC-total scores and subscales (Point-Biserial correlation test; all *P* > 0.05). Therefore, the older age of the UME students may have the more consulting experience to perform consulting skills better.

### Limitations

There are several limitations to this study. First, we did not investigate the participants’ previous experience of H&P and VR. Although we invited final-year UME students who were novices in ORL-HNS to participate, the similar Mini-CEX scores in both groups may be evidence of plateauing. Improvements in H&P skills may reach a plateau due to previously repeated training at other teaching clinics. Furthermore, the 360° VR video learning model differed from the 2D VR video learning module in visual angle, immersion, and perspective; VR beginners may need more practice to engage in 360° VR video learning proficiently. Second, we evaluated H&P skills by directly observing student-patient interactions. However, every real patient has different medical problems and needs special H&P skills and care. Although we selected patients with ORL-HNS disorders before DOPS/Mini-CEX assessments and determined the consensus of all ratings, we could not assess all domains of clinical competencies at the same time. The heterogeneities of the patients’ needs and medical information in real outpatient scenarios may have resulted in similar total scores of the Mini-CEX. However, our study reflects real-world evidence of 360° VR video learning. Third, the validated CLC questionnaire has only been verified in three studies (Naismith et al. 2015a, b, 2015a; Toy et al. 2020). Therefore, this cognitive load assessment may limit the robustness of this study. Accordingly, future research may need a more powerful tool to investigate cognitive load to optimize the instructional content and improve learning outcomes. Finally, these results were based on a short 10-min video. More follow-up studies are required to study the long-term use of 360° VR videos.

## Conclusions

360° VR video learning helped UME students to perform fundamental H&P skills as effectively as 2D VR video learning. As the 360° VR video learners acquired history taking skills as well as the 2D VR video learners during the same period, the novices who received the 360° VR video learning could perform examinations of the patient’s body more efficiently. By incorporating worked examples and self-explanation prompts, the 360° VR and 2D VR video learners experienced acceptable total cognitive loads. These findings may inspire the design of 360° VR video-based learning protocols to increase the interactive content and control intrinsic cognitive load, thereby enhancing the procedural skills of physical examination.

## Supplementary Information

Below is the link to the electronic supplementary material.

Supplementary file1 (DOCX 287 kb)

Supplementary file2 (DOCX 24 kb)

## Data Availability

The datasets generated and analyzed during the current study are not publicly available due to the privacy of the individuals who participated in the study but are available from the corresponding author on reasonable request.
