# Supplementary material for: Comparison of the effect of 360° versus two-dimensional virtual reality video on history taking and physical examination skills learning among undergraduate medical students: a randomized controlled trial
Source: Virtual Real. 2022 Aug 16;27(2):637–50. doi: 10.1007/s10055-022-00664-0 (PMC9379871; doi:10.1007/s10055-022-00664-0)
Supplement: Supplementary file 1 — Supplementary file1 (DOCX 287 kb) [file 10055_2022_664_MOESM1_ESM.docx]

**Title**

**Comparison of the effect of 360° versus two-dimensional virtual reality video on history taking and physical examination skills learning among undergraduate medical students: A randomized controlled trial**

Yi-Ping Chao, Chung-Jan Kang, Hai-Hua Chuang, Ming-Ju Hsieh, Yu-Che Chang, Terry B.J. Kuo, Cheryl C.H. Yang, Chung-Guei Huang, Tuan-Jen Fang, Hsueh-Yu Li, Li-Ang Lee

**==============================**

**Supplement 1. Study Protocol**

**Title**

**Heart Rate Variability and Cognitive Load on Image-Based Virtual Reality Instructional Design in Otolaryngology**

**Study Protocol**

**Version 1.0**

**Date April 2, 2018**

**Principal Investigator**

***Name:* Li-Ang Lee**

***Title:*** *Associate Professor*

***Department:*** *Department of Otorhinolaryngology, Head and Neck Surgery*

***Institute:*** *Linkou Chang Gung Memorial Hospital, Taoyuan City 33305, Taiwan, ROC*

**Official Title:** A Study of Heart Rate Variability and Cognitive Load on Image-Based Virtual Reality Instructional Design in Otolaryngology

**Program No.:** MOST-106-2511-S-182A-003-MY2 and 108-2511-H-182A-001-

**IRB No.:** 201601821B0

**Principal Investigator(s):** Li-Ang Lee

**Co-Investigators:** Tuan-Jen Fang, Yi-Ping Chao, Chung-Guei Huang, Hsueh-Yu Li, Li-Jen Hsin, Chung-Jan Kang

**Duration of Approval:** From 2017/08/01 TO 2020/07/31

**Approved Protocol:** 2016/12/21 Version1

**Approved Informed Consent:** 2017/01/13 Version2

**Date of Approval:** 2017/01/17

**Chairman:** Tsang-Tang Hsieh, M.D., Institutional Review Board, Chang Gung Medical Foundation

**Note:** The study has been registered on the ClinicalTrials.gov (Clinical Trials.gov Identifier: NCT03501641).

**First Submitted Date:** April 2, 2018

1. **Study Detailed Description**
   1. **Background:** Workplace-based assessments, such as mini-clinical evaluation exercise (mini-CEX), direct observation of procedural skills (DOPS), and milestones, target the highest level of clinical competence and collect information about doctors' performance in their normal practice. Recent advances in virtual reality (VR) simulation, lowering the complex of learning task and the cognitive load (CL) of the learner, make this novel technology well suited for the initial training of novices. Reduced heart rate variation (HRV), an indicator of stress, is associated with decreased cognitive efficiency in health. Accordingly, this study hypothesizes that VR-based instruction can help novices to increase HRV, decrease CL, and then improve their outcomes of workplace-based assessments.
   2. **Aims:** This prospective study aims (1) to design an image-based VR (IBVR) instruction for clinical medical education, (2) to compare HRV and CL in traditional video-based (VB) learning and novel IBVR-based learning, (3) to compare outcomes of mini-CEX, DOPS, and milestone between two modules, (4) to perform a qualitative evaluation of IBVR-based learning using a 360-degree video review, and (5) to assess various levels of novice learners (undergraduate medical students and postgraduate year residents) for their participation in and acceptance of this novel IBVR-based learning.
   3. **Study Designs:** This 3-year study includes two parts: (1) Design a VR-based curriculum including fundamental otolaryngological skills, and (2) Validation of an IBVR-based history taking and physical examination (H&P) learning activity using a blinded randomized, parallel-controlled trial in convenience-sampled novice undergraduate medical students and postgraduate year residents.
   4. **Materials and Methods:** This study will be implemented between August 1, 2017 and July 31, 2020. Firstly, a VR-based curriculum will be designed including H&P using the analysis, design, development, implementation, and evaluation model and a modified Delphi approach. VB module and IBVR module of the same contexts will be developed. Secondly, an IBVR-based H&P learning activity will be validated by measuring CL questionnaires, reaction time, HRV, mini-CEX, DOPS, milestones, global satisfaction scale and AttrakDiff2 questionnaires (n = 64). Age, sex, and cognitive style (determined by the Group Embedded Figures Test) of both groups are matched. Differences in variables of interests will be statistically analyzed.
2. **Study Design**
   1. **Study Type:** Interventional (Clinical Trial)
   2. **Actual Enrollment:** 64 participants
   3. **Allocation:** Randomized
   4. **Intervention Model:** Parallel Assignment
   5. **Intervention Model Description:** This randomized controlled trial will recruit 64 subjects who are novices in otolaryngology at an academic teaching hospital. The cognitive style of the participants is assessed using the Group Embedded Figures Test. The subjects are randomly assigned (1:1) to a novel image-based virtual reality group and conventional video-based group matched by age, sex, and cognitive style.
   6. **Masking:** Double (Investigator, Outcomes Assessor)
   7. **Masking Description:** Blinding to the purpose of the study during recruitment is maintained to minimize preparation bias. After randomization, the participants are unblinded and use image-based virtual reality model or conventional video-based model on a virtual reality eyeglass in an ordinary office environment for 10 minutes. Heart rate variation will be monitored during learning and cognitive load will be assessed by the participants. Subsequently, each participant will perform history taking and physical examination for a real patient in a teaching clinic. The competence will be evaluated using the mini-clinical evaluation exercise, direct observation of procedural skills (DOPS), and milestone assessments. Investigators and outcome assessors are blinded to their randomization and learning models.
   8. **Primary Purpose:** Health Services Research
   9. **Official Title:** A Study of Heart Rate Variability and Cognitive Load on Image-Based Virtual Reality Instructional Design in Otolaryngology
   10. **Actual Study Start Date:** April 24, 2018
   11. **Actual Primary Completion Date:** July 31, 2020
   12. **Actual Study Completion Date:** October 31, 2020
3. **Arms and Interventions**

| **Arm** | **Intervention/treatment** |
| --- | --- |
| *Experimental:* image-based virtual reality learning  The participants will undergo 10-minute image-based virtual reality learning for history taking and physical examination of otolaryngology. | *Other:* image-based virtual reality learning  The participants will use a whole-view, 360 degree image-based virtual reality to learn a competence of history taking and physical examination for otolaryngology and they can see the response of the staffs and standard patients (including image, voice, face expression, movement, ... etc). |
| *Active Comparator:* video-based learning  The participants will undergo 10-minute video-based learning for history taking and physical examination of otolaryngology. | *Other:* video-based learning  voice of the staffs and standard patients. |

1. **Outcome Measures**
   1. **Primary Outcome Measures:**
      1. Direct observation of procedural skills (DOPS) [Time Frame: DOPS will be assessed immediately after intervention.] Procedural skills of history taking and physical examination for otolaryngology will be evaluated using a DOPS assessment immediately after a 10-minute mini-clinical exercise. This assessment uses ten items (indication, explanation, preparation, sites, aseptic/safe procedure, completeness, request for help, recording, interpretation, and global assessment) with 10 (1-10) Likert scales (range: 10-100).
   2. **Secondary Outcome Measures:**
      1. Mini-clinical evaluation exercise (mini-CEX) [Time Frame: The mini-CEX will be assessed immediately after intervention.] Skills of history taking and physical examination for otolaryngology will be evaluated using a mini-CEX assessment immediately after a mini-clinical exercise. The Mini-CEX is a 10-minute direct observation assessment. This assessment uses seven items (interview, physical examination, professionalism, clinical diagnosis, explanation, efficiency, and global assessment) with 9 (1-9) Likert scales (range: 7-63).
      2. Milestones [Time Frame: The milestones will be assessed immediately after intervention.] Overall competence of history taking and physical examination for otolaryngology will be immediately evaluated using an assessment of milestones after a 10-minute mini-clinical exercise. This assessment uses five Likert-type levels (basic, focuses, appropriate, accurate, professional) (range: 1-5).
      3. Cognitive Load Scale (CLS) [Time Frame: The CLS will be assessed immediately after intervention.] Cognitive load of the specific intervention will be immediately evaluated using the Paas CLS questionnaire after an intervention. The Paas CLS questionnaire is a single-item measure of total cognitive load. Participants are asked to rate the perceived intensity of their mental effort with 9 Likert scales (1 = very, very low mental effort; 9 = very, very high mental effort).
      4. Task Load Index (TLX) [Time Frame: The TLX will be assessed immediately after intervention.] Task Load of the specific intervention will be evaluated using the National Aeronautics and Space Administration TLX questionnaire after an intervention. The questionnaire has six subscales: mental demand; physical demand; temporal demand; performance; effort, and frustration. Participants are asked to indicate the level of each dimension by making a mark on a visual analogue scale with a 21-point Likert scale (range: 0-20). Total cognitive load is interpreted as the sum of the six subscales (range: 0-120).
      5. Cognitive Load Component (CLC) [Time Frame: The CLC will be assessed immediately after intervention.] Component of cognitive load of the specific intervention will be immediately evaluated using the CLC questionnaire after an intervention. The CLC questionnaire has six subscales: difficulty, complexity, clarity, relevance, focus, and learning with 5 Likert scales (range: 6-30).
      6. Bidirectional feedback and reflection [Time Frame: The bidirectional feedback and reflection will be assessed immediately after intervention.] Bidirectional feedback and reflection will be immediate evaluated using a qualitative questionnaire after an intervention.
      7. Global Satisfaction Scale (GSS) [Time Frame: The GSS will be assessed immediately after intervention.] Global satisfaction of the specific intervention will be immediately evaluated using the GSS questionnaire with 11 Likert scales (range: 0-10) after an intervention.
      8. Pragmatic Quality (PQ) [Time Frame: The PQ will be assessed immediately after intervention.] PQ of the specific intervention will be immediately evaluated using the AttrakDiff2 questionnaire after an intervention. The AttrakDiff2 questionnaire contains 28 questions with 7 Likert-type scales. A PQ scale ranges from -3 to 3.
      9. Hedonic Stimulation (HQ-S) [Time Frame: The HQ-S will be assessed immediately after intervention.] HQ-S of the specific intervention will be immediately evaluated using the AttrakDiff2 questionnaire after an intervention. The AttrakDiff2 questionnaire contains 28 questions with 7 Likert-type scales. A HQ-S scale ranges from -3 to 3.
      10. Hedonic Identification (HQ-I) [Time Frame: The HQ-I will be assessed immediately after intervention.] HQ-I of the specific intervention will be immediately evaluated using the AttrakDiff2 questionnaire after an intervention. The AttrakDiff2 questionnaire contains 28 questions with 7 Likert-type scales. A HQ-I scale ranges from -3 to 3.
      11. Attractiveness (ATT) [Time Frame: The ATT will be assessed immediately after intervention.] ATT of the specific intervention will be immediately evaluated using the AttrakDiff2 questionnaire after an intervention. The AttrakDiff2 questionnaire contains 28 questions with 7 Likert-type scales. A ATT scale ranges from -3 to 3.
   3. **Other Outcome Measures:**
      1. Heart Rate Variation [Time Frame: At baseline for 2 minutes and during an intervention for 10 minutes.] Before and during an intervention, heart rate is continuously recorded. Heart rate variations will be calculated at baseline and during the specific intervention.
      2. Reaction time [Time Frame: At baseline, the 5th minute and the 10th time.] The dual-task paradigm with reaction time measurement is manually measured (seconds).
2. **Eligibility Criteria:**

| Ages Eligible for Study: | 20 Years to 65 Years   (Adult, Older Adult) |
| --- | --- |
| Sexes Eligible for Study: | All |
| Accepts Healthy Volunteers: | Yes |

**Criteria**

- 1. Inclusion Criteria:
     1. Age > 20 years old;
     2. Undergraduate medical students (defined as the last year of medical school training) and postgraduate year residents (defined as the first year after graduation).
  2. Exclusion Criteria:
     1. Pregnant, hypertension, recent motion sickness, inner ear infections or claustrophobia, recent surgery, pre-existing binocular vision abnormalities, heart conditions or epileptic symptoms;
     2. Declining to participate.
     3. Contacts and Locations

1. **Locations** Linkou Chang Gung Memorial Hospital, Taoyuan City, Taiwan, 33305
2. **Sponsors and Collaborators** Chang Gung Memorial Hospital
3. **Investigators**
   1. Principal Investigator: Li-Ang Lee, MD, MSc Linkou Chang Gung Memorial Hospital, Taoyuan City 33305, Taiwan, ROC
4. **Keywords provided by Chang Gung Memorial Hospital:**

cognitive load, competence, heart rate variation, learning, otolaryngology, video, virtual reality

1. **Additional relevant MeSH terms:**

Tachycardia, Arrhythmias, Cardiac, Heart Diseases, Cardiovascular Diseases, Cardiac Conduction System Disease, Pathologic Processes

**Detailed Protocol**

1. **Introduction**
   1. **Background**

Since 2009, the Accreditation Council for Graduate Medical Education (ACGME) requires that all residency programs have an effective plan for assessing their residents’ performance in all six core competencies.^1^ For further allowing programs to track the educational progress of their residents– culminating in readiness to enter unsupervised practice, and using objective data aggregated to the program level to report on otolaryngology programs' educational effectiveness for accreditation purposes, the ACGME and the American Board of Otolaryngology developed the Otolaryngology Milestones in 2012.^2^ Other workplace-based assessments, such as mini-clinical evaluation exercise (mini-CEX), and direct observation of procedural skills (DOPS), target the highest level of clinical competence and collect infor- mation about doctors' performance in their normal practice. Most of these milestones applied that ‘Obtains basic his- tory and physical exami- nation.’ as ‘Level 1 (The resident demon- strates mile- stones expected of an incoming resident.)’ and ‘Obtains focused history and physical, including comprehensive head and neck exam, neck and cranial nerve exam;’ as ‘Level 2 (The resident is advancing and demonstrates additional milestones, but is not yet performing at a mid-residency level.)’. This definition highlights the importance of precise history taking & physical examination (H&P).

The history in otolaryngology, as with all other branches of medicine and surgery, is of the utmost importance. The information gleaned during this part of the consultation will guide one towards particular areas during the examination and indicate which investigations may be appropriate; this is essential if the doctor is to come to the correct diagnosis. The interactions during history-taking form the foundation of a strong doctor-patient relationship. This is vital if any effective treatment plan offered by the doctor is to be acted upon by the patient. Although the abilities of H&P are essential for general physicians, however they may be hindered by low priority assigned to otolaryngology in the undergraduate medical education (UME) curriculum and hard to be inspected during physical examinations.^3^ A huge mismatch between this educational need and existing curricular delivery was thought to result in substantial downstream effects on managing otolaryngological problems in family medicine residents^4^ and primary care providers.^5^

For enhance skill development of H&P, commonly used methods to assess these competencies are in-training examinations, 360-degree evaluations, OSCE, surgical case logs, index case analysis, web-based instruction, outpatient clinic exposure, and oral examinations of clinical practice.^6^ Recently, technologies such as podcasts and videos with flipped classrooms, mobile technology in e-learning (M-TEL), video games, simulations (part-time trainers, integrated simulators, virtual reality [VR]), and wearable devices (google glass) are some of the techniques available to address the changing educational environment.^7^

- 1. **Virtual Reality**

VR, defined as a complex, computer-simulated environment that can provide the sensation of physical presence in places representing real or imagined worlds, opens up a vast number of additional possibilities in many domains of medical education, including surgery^8,9^ and rehabilitation.^10^ In VR simulations, the computer display simulates the physical world and user interactions are with the computer within that simulated (virtual) world. There are a number of VR programs used in medical education, such as Minimally Invasive Surgery Trainer–Virtual Reality,^11^ LINDSAY Virtual Human Project,^12^ and Second Life.^13^ Image-based VR (IBVR) system is an extensive family of advanced VR-telepresence systems and components have been developed to facilitate recording, processing, display, and interaction with audio and video signal(s) representing a scene or subject of 3-D. In recent 1 year, new camera technology such as 360-degree cameras has been successfully commercialized and fusion of images helps teachers to produce 360-degree videos more easily. VR, Mixed Reality and 360-degree videos are starting to change the shape of education. Applications, to include visual and auditory simulation, host vehicle control, remote vehicle control, video teleconferencing, and so on, are feasible applications for the above technology.^14^

Expectedly, virtual environment users often report having a sense of being present in the virtual place or a sense that the virtual object is present in their environment. This sense of presence depends on both the technological fidelity (e.g., in graphics, haptics) and the users' cognitive/ personality characteristics. Hecht and Reiner found that field-independent (FI) individuals reported higher presence ratings compared to field-dependent participants.^15^ Field dependency is defined as the degree to which a person’s perception is affected by the context of the surrounding perceptual field.^16^ The field-dependent (FD) individuals perform poorly on the Rod & Frame test, as they take the frame as a reference point for horizontal and vertical and try to rotate the rod in relation to the frame. In our recent studies that supported by the Ministry of Science and Technology (104-2511-S-182 -010 - & 105-2511-S-182A-006 -), we preliminarily found 13% of UME students were FD and 87% were FI. Although mean change of duplicated multiple choice questionnaire (MCQ) was equivalent (155% ± 126% vs. 56% ± 54%, *P* = 0.303) between FD group and FI group and mean change of duplicated multimedia situational test (MST, duplicated) of the FD group was significantly lower than that of the FI group (-20% ± 20% vs.16% ± 27%, *P* = 0.040) after a 100-minute M-TEL instructional course of emergent otorhinolaryngology-head and neck disorders. Nevertheless, global satisfaction scale (GSS) reported by FD subjects was not statistically significantly lower than that of FI subjects (5.0 ± 2.6 vs. 7.3 ± 2.1, *P* = 0.097). FI learners reported a significantly higher satisfactory to game-based learning (GBL) (8.5 ± 0.5 vs. 6.1 ± 2.4, *P* = 0.011). These findings suggested that more than 80% of UME students are FI and can perform M-TEL well, especially preferred to use the GBL module well. In contrast, as Hecht and Reiner’s findings,^15^ there was a possible linkage between FD, the sense of presence, and simulator sickness phenomenon. However, their knowledge gaining seemed not to be reduced in terms of MCQ test. Despite cognitive style can refer to the individual’s consistent attitudes for perceiving, remembering, organizing, processing, thinking, and problem solving in e-learning programs,^17^ it seemed not significantly affect UME students’ preference to use e-Learning, but the youngth generation (Y-generation) obviously liked to use the GBL module of M-TEL. When we used the AttrakDiff2 questionnaire,^18^ to be able to quantify attractive, identifiable, stimulating, and pragmatic qualities according to their learning experience, UME students felt more attractive to the M-TEL (attractive score ≥ 1.5) had a better MCQ score than those felt less attractive (< 1.5; 103% ± 84% vs. 42% ± 50%, *P* = 0.039). These findings could be explained by reduced cognitive load (CL). Two ‘FD’ students in our previously studies reported that ‘GBL confused their directions of learning and they needed very, very high intensity of mental effort to pass the games in order to learn the instructional content’. The group interview suggested the importance of cognitive style and CL overload in M-TEL.

- 1. **Cognitive Load Theory**

CL Theory is a conceptual framework relevant to activities that involve executing tasks, by focusing on the management of working memory during learning. In general, if the total CL in the learning situation results in a cognitive overload, i.e., the limits in working memory and information processing are exceeded, actual learning and skills acquisition can be impeded according to the CL theory. Theory of CL is one of the leading theories of learning and provides a theoretical framework of the cognitive architecture with the basic assumption that working memory and information processing is limited.^19^ The theory suggests 3 sources of CL in any learning situation: (1) the intrinsic load of the learning task, (2) the extraneous load provided by the learning situation, and (3) the germane load of the learning process itself.^20^ The intrinsic load is related to the task difficulty and the learner’s level of expertise. The extraneous load is related to how the task is presented and to elements that are unconnected to the goals of the task and not essential for learning. The germane load is related to the learner´s level of concentration, which is important to long-term storage of new information.^20^ Andersen *et al*. observed that the learners׳ CL in the learning situation in complex learning tasks such as learning the ear surgical procedure, should be considered because this could have implications for the organization of skills training.^21^ When the learner needs to integrate complex multisource information and psychomotor skills, learning tasks can impose an extraneous CL resulting in a cognitive overload. An efficient instructional design should aim to minimize extraneous load, to manage intrinsic load and to optimize germane load. Therefore, we can employ instructional strategies and design principles to lower the extraneous load and optimize intrinsic and germane loads, leading to more efficient learning.

Sweller et al.^20^ recommended three design principles and strategies according to theory of CL: ‘Decreasing extraneous load’, ‘Managing intrinsic load’, ‘Optimizing Germane load’, and ‘Dealing with expertise reversal effect’. When we design a health professional education for novice learners, we should consider ‘goal-free principle’, ‘worked example principle’, ‘completion principle’, ‘split attention principle’, ‘modality principle’, and ‘redundancy principle’ to reduce extraneous load. Different pedagogic approaches can also reduce the extraneous load, such as, splitting the main task in partial ones which students can complete by themselves (problem completion), providing worked examples and maintaining one integrated source of information.^22,23^ We can use ‘simple-to-complex strategy’ and ‘low- to high-fidelity strategy’ to manage intrinsic load. Intrinsic load should be managed through selection of tasks that matches learner´s level of expertise.^24^ While trying to optimize germane load, we should consider ‘variability principle’, ‘contextual interference principle’, and ‘self-explanation principle’. We may apply ‘completion strategy’, ‘fading guidance strategy’, ‘integrated to non-integrated strategy’ and ‘dual- to single-mode strategy.’ For example, when teaching students medical diagnosis, start with textual case descriptions, continue with computer-simulated patients or patients played by peers, go on to simulated patients played by actors, and end with real patients in an internship in hospital (low- to high-fidelity strategy). Germane load can be also improved with activities that provide schemata construction and automation.^22,23^

Gerjets and Scheiter^25^ further augmented CL theory and increased complexity allows CL theory to be extended to a larger range of instructional settings that are characterized by a high level of learner control (e.g., self-controlled, computer-based learning environments) CL theory provides a useful framework for analyzing instructional design features with regard to their suitability for supporting processes of schema construction and automation. This analysis is based on determining the pattern of intrinsic, germane, and extraneous cognitive load associated with a specific instructional design at a particular level of expertise. However, to extend the range of successful applications of CL theory to instructional settings that are characterized by a high level of learner control (e.g., self-controlled learning in hypertext environments), CL theory can be augmented by several variables such as configuration of teacher goals, configuration of learner goals, and learners’ processing strategies that moderate the relation between instructional design and the resulting pattern of cognitive load. These augmentations of CL theory are necessary to account for the rather weak relation between instructional design and pattern of CL that shows up in the context of self-controlled learning and may better serve as a theoretical foundation for the design of VR-based instruction.

- 1. **VR, CL, and Medical Education**

The use of simulation in health professional education has increased rapidly over the past 2 decades. VR simulation has predominantly been used to train health professionals and students for a variety of clinically related situations, especially in clinical training such as surgy^26^, endoscopy,^27^ general anesthesia,^28^ and emergency.^29^ Much desired outcomes in healthcare such as improvement of patient safety and the reduction in costs and morbidity after use of computer-enhanced training have been reported.^30^ Most of the previous studies and reviews concluded that “Because VR simulation is less complex it reduces the CL of the learner, in turn leading to better learning and making VR simulation training well suited for the initial training of novices.” However, the haptic interaction and different visual cues in VR simulation could impose additional CL compared with other training modalities. Accordingly, when we develop VR education, we should consider relevant differences in both the specific training modalities (i.e., virtual and cadaveric dissection training) and in the organization of clinical skills training in general.

Total CL has been measured with self-rating evaluation of learner’s mental effort, response time to secondary tasks, and psychophysiological measures.^20,31-35^ Instruments such as the Paas Cognitive Load Scale (CLS),^36^ the NASA Task Load Index (TLX),^37^ and the cognitive load component (CLC)^34^ have been used as measures of total cognitive load. In the dual-task paradigm, performance in a secondary-task estimates CL, e.g., by measuring reaction time in response to a visual, auditory, or tactile stimulus. Secondary-task reaction time performance has been demonstrated to detect changes in CL in surgical skills training of novices.^21,33^ However, testing predictions about the effects of different instructional design features requires sensitive and reliable measures of cognitive load. Without careful validation, the use of such measures in medical

simulation training raises significant potential concerns. For example, although they have been implemented interchangeably for measuring working memory demand, the extent to which the Paas Scale and the TLX refer to the same theoretical construct is unclear.^34^ Naismith *et al.* found that quantitative and qualitative findings supported intrinsic cognitive load as synonymous with mental effort (Paas Scale), mental demand (TLX) and task difficulty and complexity (CLC questionnaire). to separately assess intrinsic, extraneous and germane loads. Accordingly, more complete understanding of the sources of extraneous and germane cognitive loads in simulation-based training contexts is necessary to determine how best to measure and assess their effects on learning and performance outcomes.

Although VR modalities may create a digital environment, designed to resemble aspects of the real world. However, even today’s most advanced virtual technologies are still imperfect and there is always a gap between the virtual experience and the real world experiences such as visual-graphics fidelity, synchronization of multi-sensory perceptions, engineering of direct non-mediated haptic sensations. The user needs to “fill the gap” by creatively “filling in” some missing information or sensory signals, either by using cues from one sensory modality to compensate for impoverished and incomplete stimuli in another sensory modality or/and by using his own creative imagination, previous knowledge etc. to construct from the entire set of presented stimuli and his internal knowledge a complete and coherent experience.^38^ These issues can be overcome by augmented reality (AR). AR differs from VR because the focus of the interaction of the performed task lies within in the real world (AR) instead of the digital environment (VR).^39^ AR thus offers the opportunity of a digital, often interactive overlay onto a real or virtual environment. The use of AR is of real interest to medical education because they blend digital elements with the physical learning environment. However, high-end world-fixed displays with multiple surfaces and projectors can be extremely immersive but expensive. We thought that ‘IBVR technology using 360-degree videos can be a good resolution of low fidelity of VR and expensive cost of AR in simulation education.’.

- 1. **Preliminary Qualitative Evaluation of IBVR Learning**

We attempted to use 360-degree camera to record surgical skills in our dairy operative scenes. We can clear find a gap between the virtual scene and the real scene. Leaners can change the position of his head and see the operative procedure, the teacher’s response, and the surgical and anesthetic staffs’ assistance in real time and postoperative self-evaluation.

Moreover, there is an increasing trend to use simulation as an assessment tool, especially for the development of technical-based skills required during clinical practice. Notably, VR simulation-based assessment is still rare.^40-42^ Ryall *et al*. concluded that ‘Although VR simulation has now been embedded across a range of health professional education and it appears that simulation-based assessments can be used effectively; however, limitations of the previous studies included small participant numbers, poor methodological quality, and predominance of studies from medicine, which preclude any definite conclusions’.^43^ We find that VR simulation -based assessments with bidirectional feedback are very promising. One of our junior residences (R2) being watching a 360-degree 3D video of his tonsillectomy training. He completed the task well and scored 90 point by himself and got 80 point by a rater using the standard DOPS assessment. He requested that the reason that he got low scores of preoperative preparations and surgical procedure. We reviewed his procedural skills using 2D- and 3D-video records and explain the reasons at the deficient points. He also caught these points repeated by self-reviewing his 3D video. He provided some valuable positive feedbacks such as ‘Easily make a reflection and a progress of learning clinical skills in first person view.‘; ‘Learners can simulate the procedure skill using the VR technology in teacher’s view.’; ‘IBVR is a favorable instructional tool!’ Of note, we could also find the needs of CL adjustment, heart rate monitoring, and prevention from vertigo-related complications.Exactly, VR is not suitable for those who are pregnant, have high blood pressure, suffer from motion sickness, inner ear infections or claustrophobia, had any form of recent surgery, pre-existing binocular vision abnormalities, heart conditions or epileptic symptoms. Besides, taking a 10- to 15-minute break from using the VR every 10–15 minutes by removing the headset and allowing for a minimum of 30 minutes after VR using before operating any machinery have been recommended.

- 1. **Psychophysiological Reaction to IBVR Learning**

Physiological changes due to exposure to stress include increases in heart rate (HR) and blood pressure;^44^ decreased HR variability (HRV);^45^ and alterations in skin and body temperature; elevations in electrodermal response and respiratory rate.^46^ As a consequence of stress-induced sympathetic nervous system activity, HR is substantially elevated. Comparing with the extremely stressful nature of ‘Survival, Evasion, Resistance, and Escape’ training procedures (120 BPM) executed by U.S. Army school,^46^ people engaged in public speaking and mental arithmetic exercises have less pronounced HR reactions (110 BPM).^47^

The assessment of HRV has gained importance as a technique to explore the function of the autonomic nervous system and has been widely used to diagnose both psychological and physiological disorders.^48^ HRV is the beat-to-beat variation in time of consecutive heartbeats. Higher HRV characterizes a healthy person with efficient autonomic mechanisms and good adaptation ability while lower HRV is an indicator of abnormal and insufficient adaptation of the ANS.^49^ Stress likely triggered increased motivation and the recruitment of additional cognitive resources that minimize its aversive effects on task performance, but these compensatory efforts consumed resources that caused a loss of cognitive efficiency.^50^ Nevertheless, reduced HRV is suggested to be associated with harmful events in health^51^ or a greater amount of reported stress.^52^ Although there is no straightforward effect of stress on cognitive performance, and human brain does not seem defenseless against adverse effects of stressful situations. For example, stress can disrupt working memory, and, reciprocally, working memory can modulate anxious response.^53^ Learning-related stress and physical inactivity are major concerns in medical education today.

- 1. **Preliminary Quantitative Evaluation of IBVR Learning**

The current interest is in reducing learning-related stress and cognitive dysfunction, and objective methods, such as the HRV-based approach, are needed to diagnose stress symptoms. For example, the healthy UME volunteers have a median HR of 78 bpm and a median HRV of 10.13% at rest. When they watched a 15-minute teaching IBVR video, these novice learners paid much attention to the procedures of tonsillectomy and had increases in both heart rate and HRV (88 bpm & 30.99%, respectively). In contrast, median HR increased (86 bpm) but HRV reduced (7.32%) when they watched an extremely exciting VR of rolling coaster. After watching that video, they reported palpitation and vertigo, and fully recovered within 10 minutes. According to Mandrick’s recent report: ‘Higher task difficulty (high mental workload and stressful situations) degraded the performance and induced an increased tonic HR and a decreased HRV.^50^’, our preliminary findings indicated that 1) improper IBVR instructional design such as taking rolling coaster decreasing the performance; and 2) well designed VR triggering efficient autonomic mechanisms and good adaptation ability.

Because the relationship between psychology and psychophysiological reaction is not always one-to-one (e.g. one psychological operation associated with one psychophysiological reaction), but also many-to-one, one-to-many, or many-to-many,^51^ we suggest that a single psychological measure is not adequate to give a full picture of the ongoing psychological processes, particularly when both cognitive and stressful variables are manipulated. In a clinical educational study, combined heart-related measures and cognitive measure are conducted.

In last 2 years, our observations of the randomized controlled pilot study indicated that using the conscientiously designed M-TEL instructional materials, especially the GBL (interactive multimedia) module, can help the UME students and junior residents to reinforce their priori knowledge of emergent otolaryngology disorders with better satisfaction. These preliminary results support us to perform further studies on multimedia instructional design and outcome assessment. However, IBVR instructional design in otolaryngogical novice learners (core skills such as H&P, fundamental surgery), psychological and psychophysiological variables (cognitive style, CL, HRV), and assessments (milestones, mini-clinical evaluation exercise [mini-CEX], direct observation of procedural skills [DOPS], satisfaction, learning experience) need further researches. Since most medical educators have notified that ‘2016 is the year of a VR era’ and over the next few years there will be a rapid emergence of VR, many of which will have direct implications for teaching otolaryngology and other subjects.^52,53^ Accordingly, we apply a novel study of HRV and cognitive load on IBVR instructional design in otolaryngology.

1. **Objectives:**

In this prospective quasi-experimental study, the aims are:

- 1. to setup up an IBVR system for clinical medical education (H&P and basic surgeries) using the Analysis, Design, Development, Implementation, and Evaluation (ADDIE)^52^ model and a modified Delphi approach,^53^
  2. to compare CL and HRV in traditional video-based (VB) learning and novel IBVR simulation learning using the CL questionnaires and the dual-task paradigm with reaction time measurement,^21^ and a portable HR measurement under the control age, sex, and cognitive style (determined by the Group Embedded Figures Test [GEFT]),
  3. to compare outcomes (mini-CEX, DOPS, and milestone) of performance of H&P and basic surgery,
  4. to perform a qualitative evaluation in order to identify the learner’s reflection level using the 360-degree IBVR-based review of personal performance and bidirectional feedback,
  5. to assess various levels of novice learners (UME students, postgraduate year [PGY] and junior residents) for their participation in and acceptance of this novel IBVR learning compared with traditional VB learning using the GSS and AttrakDiff2^18^ questionnaire.

We will apply a systematic approach to develop a promising IBVR instructional curriculum at the end of this study.

1. **Importance:**
   1. Construction of an economical, feasible and safe IBVR-based curriculum of fundamental clinical skill such as H&P and basic surgeries for otolaryngological novice learners.
   2. Real recognition of the differences in learning performance on two verified instructional modules: VB learning and IBVR learning.
   3. Further understand effects of stress and cognition on learning outcomes of an IBVR instructional curriculum.
   4. Improvement in IBVR instructional design according to psychological and psychophysiological measurements, satisfaction, and learning experience to make progress in instructional quality.
   5. Providing for a more objective and reflective 360-degree IBVR-based assessment for advancing self-efficacy.
   6. Critically pedagogical evaluation and improvement of this educational innovation in order to implement this IBVR technology in clinical curricula to reform the current otolaryngology and medical education.

**4. Materials and Methods**

1. **Study Proposal and Reasons:**
2. **Study design**

Originally, this ***3-year study*** includes three parts: (1) Design a VR-based curriculum including fundamental otolaryngological skills such as H&P and basic surgeries; (2) Validation of an IBVR-based H&P learning activity using a blinded randomized, parallel-controlled trial in convenience-sampled novice UME and PGY learners; and (3) Validation of basic IBVR-based surgical trainings using a randomized, controlled, crossover trial in convenience-sampled novice residents. The present study will be conducting from August 1, 2017 to July 31, 2020 at a tertiary medical center–Linkou Chang Gung Memorial Hospital, Taoyuan, Taiwan. This study is approval by the Institutional Review Board of Chang Gung Medical Foundation, Taipei, Taiwan. All procedures will be carried out in accordance with the current regulations. All of the subjects need to provide written informed consent to participate in this study. With this research, something out of the ordinary is being done in their community. It is possible that if others in the community are aware that they are participating, they may ask them questions. We will not be sharing the identity of those participating in the research. The information that we collect from this research project will be kept confidential. Information about the subjects that will be collected during the research will be put away and no-one but the researchers will be able to see it. Any information about the participants will have a number on it instead of their name. Only the researchers will know what their number is and we will lock that information up with a lock and key. It will not be shared with or given to anyone except research sponsors or DSMB board.

1. **Design an IBVR-based curriculum including fundamental otolaryngological skills such as H&P and basic surgeries**

Since the recommended duration of IBVR is within 15 minutes, we choose 5 representative teaching activities that can complete in 15 minutes: H&P, ventilation tube insertion, preparation of navigation-assisted endoscopic sinus surgery, preparation of microscopic laryngeal surgery, and tracheostomy.

In the ***first-year study***, we will perform **needs assessments** of these fundamental otolaryngological skills in focused group (novice learners: 30 UME students, 20 PGY residents, and 10 junior otolaryngological residents), collection of **instructional materials** of H&P and **content validation** using the modified Delphi method in an expert panel (8 academic staffs and 2 department chiefs), **module establishment** of the VB and IBVR modules in first person view, and translate these to a **computer program**. **Outcome evaluation** using the mini-CEX for history taking, the DOPS for physical examination, the milestone assessment for level identification, the GSS questionnaire for satisfaction, and the AttrakDiff2 questionnaire for learning experience, and verify this VR-based learning tool in a **pilot study**. Meanwhile, we will measure their cognitive style using the GEFT test, CL using the CLS, the TLX, and CLC questionnaires, and the dual-task paradigm with reaction time measurement; measure HRV using a mobile HR measurement. The VB module and IBVR module consist of video and audio (non-haptic design). The main differences in these two modules the field of vision (VB: narrow view, 100-degree; IBVR; whole view, 360-degree) and involvement of staffs and patients (VB: voice; IBVR: image, voice, face expression, movement, response…, etc.). Their contexts were adjusted to the same levels by two senior investigators.

In the ***second-year study***, we will further collect instructional materials of ventilation tube insertion, preparation of navigation-assisted endoscopic sinus surgery, preparation of microscopic laryngeal surgery, and tracheostomy, and validate content, establish the VB and IBVR modules, translate these to a computer program, outcome evaluations, and verify this IBVR-based learning tool in a pilot study. We will measure their CL and measure HRV during the learning activity. We will improve our instructional design to reduce extraneous CL and increase germane CL using the CL theory and several PDCA-cycles.^57^

1. **Validation of an IBVR-based H&P learning activity using a blinded randomized, parallel-controlled trial in convenience-sampled novice UME and PGY learners**

In the ***second-year study***, we will perform a blinded randomized, parallel-controlled trial to validate the efficacy of IBVR-based learning activity among multilevel otolaryngological novice learners for H&P training at the ‘Teaching Clinics”. One of our learning objectives in Teaching Clinics is ‘The learners can precisely and proficiently perform H&P in outpatients.’. There are 4 steps in Teaching Clinics: 1) debriefing, 2) H&P, 3) assessment, and 4) feedback. VB or IBVR-based teaching for 10 minutes with monitoring HRV and immediate assessment of CL questionnaires and reaction time are implemented before receiving information and situation-based reminders of context in the first step. The participants will be randomly allocated to use a VB module or a IBVR module under the control of age, sex, and FD/FI. Then the leaners will perform H&P with video recording using a 360-degree digital camera for 10 minutes. The teachers are blinded to VB/IBVR information and need to carefully assess the prevision and proficiency of H&P, communicational skills, patient-doctor relationship…, etc. using the mini-CEX, DOPS, and milestone instruments, and prevent the patients from potential harm. Then the learners can watch their own IBVR video and feedback to and discuss with the teachers (bidirectional feedback) and provide their reflection whereas the investigators can qualitatively evaluate this IBVR-based H&P learning activity. Finally, the learners will rate their GSS and learning experience.

1. **Null Hypothesis:**
2. Mini-CEX, DOPS, and milestones scores of IBVR learning are equivalent to those of VB learning.
3. CL in terms of CLS, TLX, CLL, and reaction time of IBVR learning are equivalent to those of VB learning.
4. Stress in terms of HRV of IBVR learning are equivalent to those of VB learning.
5. Satisfaction and learning experience of IBVR learning are equivalent to those of VB learning.
6. **Participants**

For the relative small sample size of the present study, we analyzed all variables using non-parametric approaches. The sample size calculations for the trial are performed with G*Power (version 3.1.9.2; University Kiel, Germany) using the primary outcome effects (mean DOPS score; 85±10 [VB module] & 90±10 [IBVR module) in the pilot study. Using a two-tailed Wilcoxon signed-rank test for calculating the sample size (normal parent distribution; effect size, 0.50; type I error, 0.05; power, 95%), we got a sample size of 29 in each group at the minimum power of 0.95. For considering an approximate 10% drop-out rate to fulfill the criteria of intention-to-treat analysis, we needed at least 32 participants to attend this study.

The **inclusion criteria** of the present study are:

1. Age > 20 years old;
2. UME students (defined as the last year of medical school training) and PGY residents (defined as the first year after graduation).

The **exclusion criteria** are:

1. Pregnant, hypertension, recent motion sickness, inner ear infections or claustrophobia, recent surgery, pre-existing binocular vision abnormalities, heart conditions or epileptic symptoms;
2. Declining to participate.


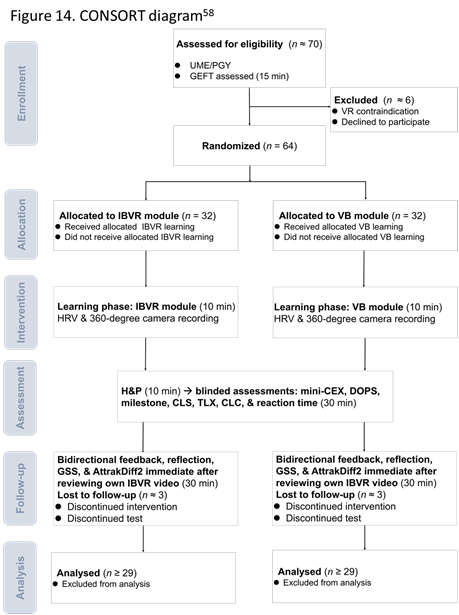


1. **GEFT (15 minute):**

We plan to recruit 64 volunteers from 70 UME participants who have listened our initial instruction of the present study and completed a 15-minute GEFT (3-minute illustration and 12-minute test). The GEFT test which is the most widely used version of pencil-and-paper tests in FD/FI investigations.^54^ The GEFT is a 25-item test that requires participants to locate and trace simple geometric figures embedded within progressively more complex ones. This test has three sections and the procedures for the administration of GEFT strictly follow the directions included in the manual (GEFT booklet pack, Mind Garden, Inc., Menlo Park, CA, USA). The first section is a practice section that contains seven simple embedded figures, and it is not scored. The time limit on this section is two minutes. The real task begins at the second set and into the third one, where the participants have to find the simple geometric figures inside two 9-itemed set within the time limit of five minutes for each of the booklet. Based on the number of correct answers given by subjects, the scores on GEFT range from 0 (the most FD) to 18 (the most FI). Those who intend to rely on external cues are less able to find the simple figures (FD), and those who hinge on internal cues are abler to find figures (FI). In this study, we will apply an English version of the GEFT that GEFT scores ≤12 are considered as ‘FD’ and those with GEFT scores >12 are regarded as ‘FI’.

1. **Randomization:**

The randomization procedure assures balanced design in age, gender, and FD/FI. Computer-generated lists of random numbers are created using Random Number Generators of SPSS software for allocation of the participants and are stratified by center with a 1:1 allocation using a fixed block size of 4 (Rv. Uniform [0, 1]) in both subgroups. The allocation sequence is concealed from the blinded researcher before instruction adhering to our computer-generated randomization protocol. Participants are randomly allocated to either IM or PPS group. Half of the patients are randomized to the IBVR group and the remaining patients are randomized to the VB group. This protocol follows the rules of the CONORT 2010.^58^

1. **Learning Material Provided (10 minutes):**

The VR-based learning is a computer program that will be developed at the Linkou-Chang Gung Memorial Hospital and Chang Gung University and. VB-based learning materials can be linked to the

participant’s headset, thus providing the pre-randomly selected VB or IBVR module to the users. Code and content for VR-based learning are kept separately. The content for the IBVR module used during the course of our study is derived from and corresponded to the VB module. Differences in the field of vision and face expression and responses of staffs and patients. During the learning period, HRV is continuously measured.

1. **HRV Measurements:**

Before (baseline, 2 minutes) and during VR-based learning (intervention, 10 minutes), HR is continuously recorded by a commercially available portable wrist pulse oximeter (3100 WristOx, Nonin Medical, Inc., Minneapolis, MN, USA) that we have used in patients with obstructive sleep apnea.^62^ The 10 sec signals before the first heartbeat are considered background noise, and the root mean square (RMS) was obtained as a baseline to distinguish heartbeats from noise. Similar to our previous studies,^63,64^ HR analysis is performed throughout each recording using a 0.25-sec time window with no overlapping data. HR is defined as the heartbeat count per minute of analysis time. In order to improve heartbeat prediction, the RMS of noise was adaptively determined at 1-sec intervals. All data were stored in a computer and processed by digital recording software (LabVIEW, National Instruments Corp., Austin, TX, USA). As mentioned above, HRV is defined as the beat-to-beat variation in time of consecutive heartbeats. HRV is acquired using a specially developed software program. To reduce the possibility of observer bias, HRV analysis is performed by a single investigator who was blinded to the clinical data.

1. **CL Measurements:**

The dual-task paradigm with reaction time measurement is manually measured (in hundredths/s) using a commercially available reaction timer (American Educational Products LLC, USA), where participants respond to an auditory cue (a beep) by pressing a pedal. Reaction time is measured before VR-based learning for baseline and 2 times during the intervention (t = 5 and 10 minute).

Three CL questionnaires are used in this study: the Paas CLS,^31^ the NASA TLX,^37^ and the Naismith CLC.^34^ The Paas CLS questionnaire is a single-item measure of total cognitive load. Participants are asked to rate the perceived intensity of their mental effort along a 9-point scale (1 = very, very low mental effort; 9 = very, very high mental effort). The NASA TLX has six subscales: mental demand; physical demand; temporal demand; performance; effort, and frustration.

Participants are asked to indicate the level of each dimension by making a mark on a visual analogue scale (range: 0–20). Total cognitive load is interpreted as the sum of the six subscales (maximum: 120 points). The Naismith CLC has six subscales to explore how different components of cognitive load relate to the Paas and TLX scales (range: 6–30). Two questions each are used to measure intrinsic, extraneous and germane load along 5-point Likert scales.

1. **Mini-CEX**

The American Board of Internal Medicine develop the mini-CEX for years. Mini-CEX is intended to facilitate formative assessment of core clinical skills. It can be used by faculty as a routine, seamless evaluation of trainees in any setting. The Mini-CEX is a 10-minute direct observation assessment or “snapshot” of a trainee-patient interaction. Faculty are encouraged to perform at least one per clinical rotation. To be most useful, faculty should provide timely and specific feedback to the trainee after each assessment of a trainee-patient encounter (range: 7–63).

1. **DOPS**

DOPS is developed by the Royal College of Physicians,^65^ and now forms part of workplace based assessments for doctors in the foundation year and those in specialist training including otolaryngology (range: 10–100). Most DOPS are completed by consultants, which is likely to be a reflection of the fact that otolaryngological trainees are well supported, with the majority of junior trainees having consultant supervision in theatre or within the theatre complex. A DOPS is specifically designed to assess procedural skills involving real patients in a single encounter. This is an important facet of DOPS which distinguishes it from other forms of assessment such as a supervisor's evaluation which relies on observation over a period of time. DOPS is a formative rather than summative tool and training for all those who are participating in DOPS assessments.

1. **Milestones:**

Milestones are knowledge, skills, attitudes, and other attributes for each of the ACGME competencies organized in a developmental framework from less to more advanced. They are descriptors and targets for resident performance as a resident moves from entry into residency through graduation. Milestones are arranged into numbered levels. Tracking from Level 1 to Level 5 is synonymous with moving from novice to expert. These levels do not correspond with post-graduate year of education. Selection of a level implies that the leaner substantially demonstrates the milestones in that level, as well as those in lower levels. Some milestone descriptions include statements about performing independently. These activities must occur in conformity to institutional and program policies. For example, a leaner who performs a procedure independently must, at a minimum, be supervised through oversight.

1. **GSS and AttrakDiff2:**

GSS score is measured using a visual analogue scale from 0 to 10 as mentioned above. AttrakDiff2 is developed as a tool by Hassenzahl’s research group to be able to quantify attractive, identifiable, stimulating, and pragmatic qualities.^18^ The tool uses 4x7 anchor scales, in total 28 questions. The anchors are presented in the form of semantic differentials and a 7-point Likert scale is employed for rating the intensity of the items. Each of the mean values of an item group creates a scale value for pragmatic quality (PQ), hedonic stimulation (HQ-S), hedonic identification (HQ-I), and attractiveness (ATT). Attributes in the PQ group describe how easy the user finds it to work with the provided program or environment. Attributes belonging to HQ-S describe factors that encourage the personal growth of users and provide stimulation to give them the opportunity to enhance their knowledge and development. The attributes falling into the HQ-I category make it possible to identify the social impact that using a product can have for users, including the “messages” that are communicated by using the evaluated product. Last, the attributes of the ATT group depict the overall experience a product has to offer to its users, that is, its attractiveness.

1. **Outcome Measurements and Statistics:**

Primary outcome is score of DOPS. Differences in scores of mini-CEX, DOPS, milestones, CLS, TLX, CLL, GSS, PQ, HQ-S, HQ-I, and ATT between IBVR module and VB module are compared using Mann-Whitney *U* test. Changes in reaction time and HRV between IBVR module and VB module are compared using Wilcoxon rank test. Percentage (%) of change ([intervention value – baseline value]/[baseline value] × 100) in reaction time as well as HRV will be calculated and compared with Mann-Whitney *U* test or Wilcoxon rank test as appropriate.

1. **Validation of basic IBVR-based surgical trainings using a randomized, controlled, crossover trial in convenience-sampled novice residents**

In the ***second-year study***, we will perform a prospective, randomized, controlled, crossover trial to validate the efficacy of IBVR-based surgical trainings in otolaryngological novice residents (R1 & R2) in the operation room. Because we trained 5–6 new residents in each year at our department (total 10–12 junior residents at Linkou–, Keelung–, and Taipei– Chang Gung Memorial Hospital), we need to use a crossover study design (sample size = 10). A crossover study is a longitudinal study in which subjects receive a sequence of different interventions in many scientific disciplines, for example psychology, education, pharmaceutical science, and medicine. Randomized, controlled crossover experiments are especially important in health care. A crossover clinical trial is a repeated measures design in which each participant is assigned to a sequence of interventions, including at least two modules. Two of our learning objectives in surgical trainings are “The residents can precisely and proficiently prepare pre-operative setup of complex surgeries” and ‘The learners can precisely and proficiently perform basic surgeries.’. Each session of 15-minute VB or IBVR-based instruction with monitoring HRV are randomly allocated to the participants before operation. Reaction time and CL questionnaires are immediately tested after learning. Then the residents will perform a formal operation with video recording using a 360-degree digital camera for 15 minutes. The teachers are blinded to VB/IBVR information need to carefully assess the prevision and proficiency of operation, communicational skills, nurse-doctor relationship, etc., using the DOPS and milestone instruments, and prevent the patients from potential harm. Then the residents can watch their own IBVR video and find their deficiency and immediately discuss with the teachers (bidirectional feedbacks). The participants need to provide their reflection in their feedbacks. They will receive the same module of 4 consecutive sessions then receive the other module subsequently. The interval between VB and IBVR of the same operation is at least 1 month (wash-out period) to minimize possible overlapping training effects.

In the ***third-year study***, the participants will complete 4 sessions of VB or IBVR surgical trainings and then switch to receive IBVR or VB surgical trainings. Finally, the participants will complete 8 sessions of basic surgical training with 2 different modules, and they will rate their GSS and learning experience. At the end of this study, group interviews will be performed to gather their feedbacks and reflections. The investigators can also qualitatively evaluate this IBVR-based surgical trainings.

1. **Null Hypothesis:**
2. DOPS and milestone scores of IBVR learning are equivalent to those of VB learning.
3.
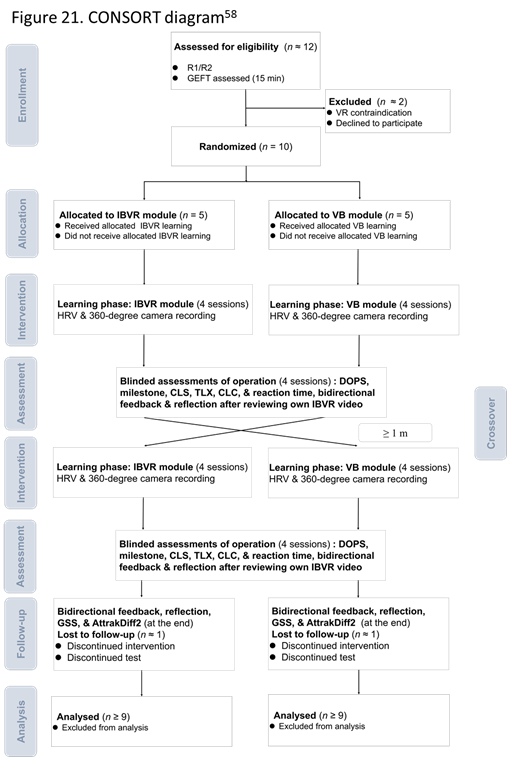
CL in terms of CLS, TLX, CLL, and reaction time of IBVR learning are equivalent to those of VB learning.
4. Stress in terms of HRV of IBVR learning are equivalent to those of VB learning.
5. Satisfaction and learning experience of IBVR learning are equivalent to those of VB learning.
6. **Participants**

The **inclusion criteria** of the present study are:

1. Age > 20 years old;
2. Junior residents (R1 and R2).

The **exclusion criteria** are:

1. Pregnant, hypertension, recent motion sickness, inner ear infections or claustrophobia, recent surgery, pre-existing binocular vision abnormalities, heart conditions or epileptic symptoms;
2. Declining to participate.
3. **GEFT (15 minute):**

We plan to recruit 10 volunteers from 12 junior residents who have listened our initial instruction of the present study and completed a 15-minute GEFT.^54^ We will apply an English version of the GEFT that GEFT scores ≤12 are considered as ‘FD’ and those with GEFT scores >12 are regarded as ‘FI’.

1. **Randomization:**

The randomization procedure assures balanced design in age, gender, and FD/FI. Computer-generated lists of random numbers are created using Random Number Generators of SPSS software for allocation of the participants and are stratified by center with a 1:1 allocation (Rv. Uniform [0, 1]). The allocation sequence is concealed from the blinded researcher before instruction adhering to our computer-generated randomization protocol. Participants are randomly allocated to either IM or PPS group. Half of the patients are randomized to the IBVR group and the remaining patients are randomized to the VB group. This protocol follows the rules of the CONORT 2010.^58^

1. **Learning Material Provided (15 minutes):**

The VR-based learning is a computer program that will be developed at the Linkou Chang Gung Memorial Hospital and Chang Gung University and. VB-based learning materials can be linked to the participant’s headset, thus providing the pre-randomly selected VB or IBVR module to the users. Code and content for VR-based learning are kept separately. The content for the IBVR module used during the course of our study is derived from and corresponded to the VB module. During the learning period, HRV is continuously measured.

1. **HRV Measurements:**

Before (baseline, 2 minutes) and during VR-based learning (intervention, 15 minutes), HR is continuously recorded as mentioned above. To reduce the possibility of observer bias, HRV analysis is performed by a single investigator who was blinded to the clinical data.

1. **CL Measurements:**

The dual-task paradigm with reaction time measurement is manually measured before VR-based learning for baseline and 3 times during the intervention (t = 5, 10 and 15 minute).

1. **DOPS**

In the third study, DOPS is specifically designed to assess procedural skills of either basic surgery or preparation of advanced surgery.

1. **Milestones:**

Milestones are arranged into numbered levels for clinical procedural skills. Tracking from Level 1 to Level 5 is synonymous with moving from novice to expert. These levels do not correspond with post-graduate year of education. Selection of a level implies that the leaner substantially demonstrates the milestones in that level, as well as those in lower levels. In case of ventilation tube insertion, level l novice learners can participate in surgical time out; level 2 novice learners can insert ear speculum and safely cleans cerumen from ear canal; level 3 middle learners can identify tympanic membrane and external auditory canal (EAC) landmark and structures; able to consistently perform appropriate myringotomy; level 4 mature learner places tympanostomy tube safely in all patients with easy anatomy and in some patients with difficult anatomy; level 5 expert places tympanostomy tube safely in patients with difficult anatomy. In our facility, the R2 learner can perform this operation independently must, at a minimum, be supervised through oversight. Levels of other operations of the present study are rated as the similar manner.

1. **GSS and AttrakDiff2:**

GSS and AttrakDiff2 are assessed at the end of each session and at the end of the third study. Each sessional GSS and learning experience can help us to improve our quality of instructional design. Overall GSS, PQ, HQ-S, HQ-I, and ATT can help us to understand the reasons of product acceptance. Such information will help us to make further progresses in instructional design of IBVR-based learning.

1. **Outcome Measurements and Statistics:**

Primary outcome is DOPS score. Because of longitudinal, crossover, repeated measures design of the present study, we use the generalized estimating equation (GEE) approach with mixed models that include random effects to facilitate analysis of data. Differences in scores of DOPS, milestones, CLS, TLX, CLL, GSS, PQ, HQ-S, HQ-I, and ATT, changes in reaction time and HRV, and % of change in reaction time and HRV are compared using the GEE test.

1. **Statistical Analysis:**

Data are analyzed using the SPSS 23.0 software (SPSS Inc., an IBM company, Chicago, IL, USA) and GraphPad Prism 5 software (GraphPad Software, Inc., San Diego, CA, USA). Statistical significance will be determined using the Mann-Whitney *U* test, Wilcoxon rank test, chi-square test, or the Fisher’s exact test as appropriate. Besides, content validity index (content validation of both VB and IBVR modules) and Cronbach’s alpha (internal consistency) will also be calculated. Correlation between variables of interests were analyzed using Spearman’s rank correlation coefficient. Besides, two-way analysis of variance (ANOVA) is used for examining the influence of two different categorical independent variables (male/female & VR-based modules; FD/FI & VR-based modules; high/low CL & VR-based modules; high/low stress & VR-based modules) on learning outcome measurements. Two-sided *P* values < 0.05 are considered statistically significant.

1. **Anticipated possible problems and resolutions**
2. We may need more eligible candidates as anticipated since we want to control age, gender, and FD/FI ratios. Fortunately, there are approximate 100 UME and PGY students from various medical colleges to be trained in our Teaching Clinics each year. Moreover, participants may withdraw from the trial or become "lost to follow-up" in most longitudinal studies of human subjects. According to our previous experience, we extremely need an experienced research assistant (RA) to recruit subjects, obtain written inform consent forms, performing experiments, and manage our experimental data. The best resolution is the MOST agree us to hide a RA in this three-year study.
3. Such as mentioned above, missing-data will be a big problem in this longitudinal study. In additional to a full-time RA, we can use an optimal method such as GEE approach to analyze the data according to the principle of the intention to treat.
4. Despite we have some experience in instructional design of mobile e-learning (M-TEL), VR is still a tremendous challenge to us because of complex program and hardware technologies and expensive instruments. Due to limited resource, we construct an experienced team including experts of medical education.
5. **Anticipated Outcomes:**
   1. **Anticipated progression of the study**

We will conduct the three-year study between August 1, 2017 and July 30, 2020. In the first year, we plan to design a VR-based curriculum including H&P and parts of basic surgeries with verification in a pilot study and validate an IBVR-based H&P learning activity using a blinded randomized, parallel-controlled trial in convenience-sampled novice UME and PGY learners (*n* = 24). In the second year, we will complete the design work of a VR-based surgical trainings with verification in a pilot study and finish the validation of an IBVR-based H&P learning activity using a randomized controlled trial (*n* = 40), and begin to recruit 10 junior residents to validate a basic IBVR-based surgical trainings using a randomized, controlled, crossover trial in convenience-sampled novice residents. In the third year, we will complete the validation of IBVR-based surgical trainings using a randomized crossover trial, and data analysis and manuscript preparation.

- 1. **Anticipated contributions to academic research, national development, and other clinical applications**

As mentioned above, there is widespread concern regarding the inadequacy of otolaryngological teaching at novice level. Improvement in M-TEL and VR-based learning of otolaryngology can drive improved learning outside the classroom. New, additional methods of teaching covering a range of 6-core competencies will be shown to be valid and reliable. Thorough medical curricula should include classroom lectures, M-TEL learning, VR simulation, clinical placement, and assessment in order to enhance otolaryngological knowledge and clinical skills for novice learners according to CL theory. This study will design an economic, feasible, safe IBVR-based instruction for self-directed learning and enhancement of self-efficacy. Moreover, identification of personally cognitive style and copping style to stress and/or CL overload can help the learners to choose a proper instructional material on the VR-based instructional system. This novel IBVR-based instruction can reform the otolaryngological learnings in novice learners. Finally, the novices can better their performance in fundamental otolaryngological skills without significant increasing their CL and stress, and ultimately improve patient safety.

- 1. **Anticipation of trainings for study researchers**

The medical teachers can be familiar with the design of the IBVR-based instruction and increase their ability of handling VR-based learning to effectively blend instructional design, CL theory, and learning objective principles. The professors of VR/AR instruction can extend research to medical education in novice level. The computer programmers can train their ability to establish an innovative IBVR-based learning tool to deliver medical education. Finally, we can combine traditional cognitive theory, learning principles, and psychophysiology in the design of new VR-based instruction.

**Reference：**

1. ACGME outcome project. **2009**. Available at: www.acgme.org. Accessed December 1, 2016.
2. Tsue TT. Developing the otolaryngology milestones. J Grad Med Educ. **2014**;6(1 Suppl 1):162-5.
3. Vinayak BC, Bates GJ. Undergraduate training in ENT - time for change? J R Soc Med **1993**;86:181.
4. Glicksman JT, Brandt MG, Parr J, Fung K. Needs assessment of undergraduate education in otolaryngology among family medicine residents. J Otolaryngol Head Neck Surg **2008**;37:668-675.
5. Hu A, Sardesai MG, Meyer TK. A need for otolaryngology education among primary care providers. Med Educ Online. **2012**;17:17350.
6. Stewart CM, Masood H, Pandian V, Laeeq K, Akst L, Francis HW, Bhatti NI. Development and pilot testing of an objective structured clinical examination (OSCE) on hoarseness. Laryngoscope. **2010**;120(11):2177-82.
7. Guze PA. Using Technology to Meet the Challenges of Medical Education. Trans Am Clin Climatol Assoc. **2015**;126:260-70.
8. Evans CH, Schenarts KD. Evolving Educational Techniques in Surgical Training. Surg Clin North Am. **2016**;96(1):71-88.
9. Maertens H, Madani A, Landry T, Vermassen F, Van Herzeele I, Aggarwal R. Systematic review of e-learning for surgical training. Br J Surg. **2016**;103(11):1428-37.
10. Rizzo A, Kim G. A SWOT analysis of the field of VR rehabilitation and therapy. Presence. **2005**;14:119–146.
11. McCloy R, Stone R. Virtual reality in surgery. BMJ **2001**;323(7318):912–5.
12. Nicholson DT, Chalk C, Funnell WR, Daniel SJ. Can virtual reality improve anatomy education? A randomised controlled study of a computer-generated three-dimensional anatomical ear model. Med Educ **2006**;40(11):1081–7.
13. Wiecha J, Heyden R, Sternthal E, Merialdi M. Learning in a virtual world: experience with using second life for medical education. J Med Internet Res **2010**;12(1):e1.
14. Ritchey KJ. "Image-based panoramic virtual reality system", Proc. SPIE 1668, Visual Data Interpretation, 2 (June 1, **1992**); doi:10.1117/12.59650.
15. Hecht D, Reiner M. Field dependency and the sense of object-presence in haptic virtual environments. Cyberpsychol Behav. **2007**;10(2):243-51.
16. Witkin, H.A., & Goodenough, D.R. Cognitive styles: essence and origins. Field dependece and independence. New York: International Universities Press; **1981**.
17. Sozcu OF. The relationships between cognitive style of field dependence and learner variables in e-learning instruction. Turkish Online J Dist Educ **2014**;15:10.
18. Hassenzahl M, Burmester M, Koller F. Der User Experience (UX) auf der Spur: Zum Einsatz von www.attrakdiff.de. In: Brau H, Diefenbach S, Hassenzahl M, Koller F, Peissner M, Röse K, editors. Usability Professionals. Stuttgart: German Chapter der Usability Professionals Association; **2008**:78-82.
19. Sweller J. Cognitive load during problem solving: effects on learning. Cogn Sci. **1988**;12(2):257-285.
20. van Merriënboer JJ, Sweller J. Cognitive load theory in health professional education: design principles and strategies. Med Educ. **2010**;44(1):85-93.
21. Andersen SA, Mikkelsen PT, Konge L, Cayé-Thomasen P, Sørensen MS. Cognitive Load in Mastoidectomy Skills Training: Virtual Reality Simulation and Traditional Dissection Compared. J Surg Educ. **2016**;73(1):45-50.
22. Young JQ, van Marrienboer J, Durning S, Ten Cate O. AMEE Guide no. 86: Cognitive Load Theory: implications for medical education. Med Teach. **2014**; 36(5): 371-384.
23. Brunken R, Plass JL, Leutner D. Direct Measurement of Cognitive Load in Multimedia Learning. Educ Psychol. **2003**; 38(1): 53-61.
24. Kalyuga S. Knowledge elaboration: A cognitive load perspective. Learning and Instruction. **2009**; 19:402-410.
25. Gerjets P, Scheiter K. Goal Configurations and Processing Strategies as Moderators Between Instructional Design and Cognitive Load: Evidence From Hypertext-Based Instruction. Educ Psychol **2003**;38(1):33-41
26. Maertens H, Madani A, Landry T, Vermassen F, Van Herzeele I, Aggarwal R. Systematic review of e-learning for surgical training. Br J Surg. **2016**;103(11):1428-37.
27. Harpham-Lockyer L, Laskaratos FM, Berlingieri P, Epstein O. Role of virtual reality simulation in endoscopy training. World J Gastrointest Endosc. **2015**;7(18):1287-94.
28. Potapov AF, Matveev AS, Ignatiev VG, Ivanova AA, Aprosimov LA. SIMULATION OF GENERAL ANESTHESIA ON THE "SIMMAN 3G" AND ITS EFFICIENCY. Wiad Lek. **2015**;68(4):565-6.
29. de Araujo Guerra Grangeia T, de Jorge B, Franci D, Martins Santos T, Vellutini Setubal MS, Schweller M, de Carvalho-Filho MA. Cognitive Load and Self-Determination Theories Applied to E-Learning: Impact on Students' Participation and Academic Performance. Cognitive Load and Self-Determination Theories Applied to E-Learning: Impact on Students' Participation and Academic Performance. PLoS One. **2016**;11(3):e0152462.
30. Graafland M, Schraagen JM, Schijven MP. Systematic review of serious games for medical education and surgical skills training. Br J Surg. **2012**;99(10):1322–1330.
31. Paas F. Training strategies for attaining transfer of problem solving skills in statistics: a cognitive load approach. J Educ Psychol. **1992**; 84: 429-34.
32. Brünken R, Plass JL, Leutner D. Direct measurement of cognitive load in multimedia learning. Educ Psychol. **2003**;38(1):53-61.
33. Haji FA, Khan R, Regehr G, Drake J, de Ribaupierre S, Dubrowski A. Measuring cognitive load during simulation-based psychomotor skills training: sensitivity of secondary-task performance and subjective ratings. Adv Health Sci Educ Theory Pract. **2015**;20(5):1237-53.
34. Naismith LM, Cheung JJH, Ringsted C, Cavalcanti RB. Limitations of subjective cognitive load measures in simulation-based procedural training. Med Educ. **2015**; 49:805-814.
35. Leppink J, Paas F, Van der Vleuten CPM, Van Gog T, Van Merriënboer JJG. Development of an instrument for measuring different types of cognitive load. Behav Res Methods. **2013**;45(4):1058-72.
36. Leppink J, Paas F, van Gog T, van der Vleuten CPM, van Merriënboer JJG. Effects of pairs of problems and examples on task performance and different types of cognitive load. Learn Instr **2014**;30:32-42.
37. Hart SG, Staveland LE. Development of NASA-TLX (Task Load Index): results of empirical and theoretical research. In: Hancock PA, Meshkati N, eds. Human Mental Workload. Amsterdam: North Holland Press **1988**;139-183.
38. Biocca F, Kim J, Choi Y. Visual touch in virtual environments: an exploratory study of pres- ence, multimodal interfaces, and cross-modal sensory illusions. Presence: Teleoperators & Virtual Environments **2001**;10:247-266.
39. Barsom EZ, Graafland M, Schijven MP. Systematic review on the effectiveness of augmented reality applications in medical training. Surg Endosc. **2016**;30(10):4174-83.
40. Grantcharov TP, Carstensen L, Schulze S. Objective assessment of gastrointestinal endoscopy skills using a virtual reality simulator. JSLS. **2005**;9(2):130-3.
41. Lipner RS1 Messenger JC, Kangilaski R, Baim DS, Holmes DR Jr, Williams DO, King SB 3rd. A technical and cognitive skills evaluation of performance in interventional cardiology procedures using medical simulation. Simul Healthc. **2010**;5(2):65-74.
42. Bick JS, Demaria S Jr, Kennedy JD, Schwartz AD, Weiner MM, Levine AI, Shi Y, Schildcrout JS, Wagner CE. Comparison of expert and novice performance of a simulated transesophageal echocardiography examination. Simul Healthc. **2013**;8(5):329-34.
43. Ryall T, Judd BK, Gordon CJ. Simulation-based assessments in health professional education: a systematic review. J Multidiscip Healthc. **2016**;9:69-82.
44. Kumar R, Goel N, Current status of cardiovascular risk due to stress, Int J Health. **2007**;7(1):1-14.
45. Teisala T, Mutikainen S, Tolvanen A, Rottensteiner M, Leskinen T, Kaprio J, Kolehmainen M, Rusko J, Kujalaet UM, Associations of physical activity, fitness, and body composition with heart rate variability–based indicators of stress and recovery on workdays: a cross-sectional study. J Occ Med Toxicol. **2014**;9:16
46. Lieberman HR, Farina EK, Caldwell J, Williams KW, Thompson LA, Niro PJ, Grohmann KA, McClung JP. Cognitive function, stress hormones, heart rate and nutritional status during simulated captivity in military survival training. Physiol Behav. **2016**;165:86-97.
47. Kudielka BM, Buske-Kirschbaum A, Hellhammer DH, Kirschbaum C. Differential heart rate reactivity and recovery after psychosocial stress (TSST) in healthy children, younger adults, and elderly adults: the impact of age and gender. Int J Behav Med. **2004**;11(2):116-21.
48. Dishman RK, Nakamura Y, Garcia ME, Thompson RW, Dunn AL, Blair SN: Heart rate variability, trait anxiety, and perceived stress among physically fit men and women. Int J Psychophysiol. **2000**, 37(Suppl 2):121–133.
49. Vanderlei LCM, Pastre CM, Hoshi RA, Carvalho TD, Godoy MF: Basic notions of heart rate variability and its clinical applicability. Rev Bras Cir Cardiovasc. **2009**, 24(Suppl 2):205–217.
50. Mandrick K, Peysakhovich V, Rémy F, Lepron E, Causse M. Neural and psychophysiological correlates of human performance under stress and high mental workload. iol Psychol. **2016**;121(Pt A):62-73.
51. Zhou F, Qu X, Helander MG, Jiao JR. Affect prediction from physiological measures via visual stimuli. Int J Human-Comput St. **2011**;69(12):801–819.
52. Arora A, Lau LY, Awad Z, Darzi A, Singh A, Tolley N. Virtual reality simulation training in Otolaryngology. Int J Surg. **2014**;12(2):87-94.
53. Piromchai P, Avery A, Laopaiboon M, Kennedy G, O'Leary S. Virtual reality training for improving the skills needed for performing surgery of the ear, nose or throat. Cochrane Database Syst Rev. **2015**;(9):CD010198.
54. Morrison GR, Ross SM, Kemp JE, Kalman H. Designing Effective Instruction, 7th Edition. Wiley Inc., Hoboken, NJ, U.S.A., **2013.**
55. Shore EM, Lefebvre GG, Husslein H, Bjerrum F, Sorensen JL, Grantcharov TP. Designing a Standardized Laparoscopy Curriculum for Gynecology Residents: A Delphi Approach. J Grad Med Educ. **2015**;7(2):197-202.
56. Chen S. A cognitive model for non–linear learning in hypermedia programmes. Br J Educ Techno.l **2002**;33(4):449-60.
57. Jin H, Huang H, Dong W, Sun J, Liu A, Deng M, Dirsch O, Dahmen U. Preliminary experience of a PDCA-cycle and quality management based training curriculum for rat liver transplantation. J Surg Res. **2012**;176(2):409-22.
58. Moher D, Hopewell S, Schulz KF, Montori V, Gøtzsche PC, Devereaux PJ, Elbourne D, Egger M, Altman DG. CONSORT 2010 explanation and elaboration: updated guidelines for reporting parallel group randomised trials. BMJ. **2010**;340:c869.
59. Pumprla J, Howorka K, Groves D, Chester M, Nolan J: Functional assessment of heart rate variability: physiological basis and practical applications. Int J Cardiol. **2002**, 84(Suppl 1):1–14.
60. Chandola T, Heraclides A, Kumari M: Psychophysiological biomarkers of workplace stressors. Neurosci Biobehav Rev. **2010**, 35(Suppl 1):51–57.
61. Schoofs D, Wolf OT, Smeets T. Cold pressor stress impairs performance on working memory tasks requiring executive functions in healthy young men. Behav Neurosci. **2009**;123(5):1066-75.
62. Chen WC, **Lee LA** (co-first), Chen NH, Fang TJ, Huang CG, Cheng WN, Li HY. Treatment of snoring with positional therapy in patients with positional obstructive sleep apnea syndrome. Sci Rep. **2015**;5:18188.
63. Lee GS, **Lee LA** (co-first), Wang CY, Chen NH, Fang TJ, Huang CG, Cheng WN, Li HY. The Frequency and Energy of Snoring Sounds Are Associated with Common Carotid Artery Intima-Media Thickness in Obstructive Sleep Apnea Patients. Sci Rep. **2016**;6:30559.
64. **Lee LA**, Lo YL, Yu JF, Lee GS, Ni YL, Chen NH, Fang TJ, Huang CG, Cheng WN, Li HY. Snoring Sounds Predict Obstruction Sites and Surgical Response in Patients with Obstructive Sleep Apnea Hypopnea Syndrome. Sci Rep. **2016**;6:30629.
65. Norcini JJ, McKinkley DW. Assessment methods in medical education. Teach Teach Educ. **2007**; 23: 239-250.
