# Supplementary material for: Comparison of the effect of 360° versus two-dimensional virtual reality video on history taking and physical examination skills learning among undergraduate medical students: a randomized controlled trial
Source: Virtual Real. 2022 Aug 16;27(2):637–50. doi: 10.1007/s10055-022-00664-0 (PMC9379871; doi:10.1007/s10055-022-00664-0)
Supplement: Supplementary file 2 — Supplementary file2 (DOCX 24 kb) [file 10055_2022_664_MOESM2_ESM.docx]

**Title**

**Comparison of the effect of 360° versus two-dimensional virtual reality video on history taking and physical examination skills learning among undergraduate medical students: A randomized controlled trial**

Yi-Ping Chao, Chung-Jan Kang, Hai-Hua Chuang, Ming-Ju Hsieh, Yu-Che Chang, Terry B.J. Kuo, Cheryl C.H. Yang, Chung-Guei Huang, Tuan-Jen Fang, Hsueh-Yu Li, Li-Ang Lee

**==============================**

**Supplement 2. Instructional Content.**

For educating the skills of history taking and physical examination, the investigators designed a 10-minute instructional video, including essential knowledge and procedural skills of H&P according to the guidelines of the American Board of Otolaryngology^1^, which was developed using analysis, design, development, implementation, and evaluation models^2^.

The instructional content of history taking included:

1. Protecting yourself while facing an actual patient during a teaching clinic.
2. Obtaining a previous medical history from the Healthcare /Hospital Information System.
3. The general framework for history taking.
4. Starting with the opening greeting and actively listen to the patient.
5. Asking the patient with open questions, questions with options, or leading questions.
6. Summarizing and confirming the history of the patient.

The instructional content of physical examination included:

1. Using traditional instruments to perform a physical examination of the head and neck.
2. The indications, relevant anatomy, and procedural techniques.
3. Obtaining agreement
4. Preparing a physical examination of the head and neck.
5. Determining the examination areas.
6. Perform a physical examination safely.
7. Seeking helps.
8. Explaining the examination findings.

**References.**

1. Tsue TT. Developing the otolaryngology milestones. *J Grad Med Educ.* 2014;6(1 Suppl 1):162-165.

2. Morrison GR, Ross SM, Kalman HK, Kemp JE. *Designing Effective Instruction.* 7th Edition ed. Hoboken, NJ: Wiley Inc.; 2013.
